# Modeling and mitigation of high-concentration antibody viscosity through structure-based computer-aided protein design

**DOI:** 10.1371/journal.pone.0232713

**Published:** 2020-05-07

**Authors:** James R. Apgar, Amy S. P. Tam, Rhady Sorm, Sybille Moesta, Amy C. King, Han Yang, Kerry Kelleher, Denise Murphy, Aaron M. D’Antona, Guoying Yan, Xiaotian Zhong, Linette Rodriguez, Weijun Ma, Darren E. Ferguson, Gregory J. Carven, Eric M. Bennett, Laura Lin

**Affiliations:** BioMedicine Design, Pfizer Inc, Cambridge, Massachusetts, United States of America; University of Lincoln, UNITED KINGDOM

## Abstract

For an antibody to be a successful therapeutic many competing factors require optimization, including binding affinity, biophysical characteristics, and immunogenicity risk. Additional constraints may arise from the need to formulate antibodies at high concentrations (>150 mg/ml) to enable subcutaneous dosing with reasonable volume (ideally <1.0 mL). Unfortunately, antibodies at high concentrations may exhibit high viscosities that place impractical constraints (such as multiple injections or large needle diameters) on delivery and impede efficient manufacturing. Here we describe the optimization of an anti-PDGF-BB antibody to reduce viscosity, enabling an increase in the formulated concentration from 80 mg/ml to greater than 160 mg/ml, while maintaining the binding affinity. We performed two rounds of structure guided rational design to optimize the surface electrostatic properties. Analysis of this set demonstrated that a net-positive charge change, and disruption of negative charge patches were associated with decreased viscosity, but the effect was greatly dependent on the local surface environment. Our work here provides a comprehensive study exploring a wide sampling of charge-changes in the Fv and CDR regions along with targeting multiple negative charge patches. In total, we generated viscosity measurements for 40 unique antibody variants with full sequence information which provides a significantly larger and more complete dataset than has previously been reported.

## Introduction

Use of antibodies as therapeutics is an increasing trend, with over 80 currently approved in the United States and/or Europe. [1] Antibodies are attractive therapeutics, as they offer greater specificity to their antigens than small molecules, in addition to significant increases in serum half-life and often favorable activity such as effector functions. [2] However, due to their biophysical properties and large size, oral administration is not possible and dosing is mainly limited to intravenous and subcutaneous (SC) injection. [3] In many clinical and commercial settings, the latter is often preferred owing to ease of use for the patient, but imposes a volume limit of 1 to 2mL per injection. [4–6] When a large dose is required the volume limit necessitates highly concentrated protein solutions up to 150 to 180 mg/ml. At these concentrations, biophysical properties associated with self-interaction such as aggregation, solubility and viscosity of antibodies can be limiting factors. For a successful SC administration of an antibody therapeutic with optimal syringeability and minimal pain, the preferred viscosity of the material is lower than 20 centipoise (cP) at the desired concentration. [7] In addition, concentrations higher than the projected dose are often required during the manufacturing process to enable buffer exchange and formulation, and excess viscosity can lead to clogged filters and loss of material. [6]

Strategies for mitigation of high-concentration viscosity include optimization of the formulations, optimization of the sequence or screening of candidate molecules for optimal properties. [8] Formulation optimizations include varying the buffer, pH, salt concentrations and use of excipients. [9–14] Moving away from standard platform conditions often requires additional analytical methods development and stability monitoring specifically tailored for each antibody, resulting in program delay and prolonged cycle time. Sequence optimization in principle could help identify variants with improved viscosity profiles in platform formulation conditions, although many such examples of viscosity reduction have proven detrimental to affinity or activity. [15–18]

Rational optimization of antibodies requires understanding, and the ability to predict, the structure/function relationship. Several studies in the literature have tried to address viscosity via modification of surface charge and hydrophobicity. Small reductions in viscosity (<2 fold) have been achieved through charge change mutations without much loss in activity. Nichols et al. reduced viscosity from 16.9 to 13.2 cP at 103 mg/ml mAb concentration through the removal of a negative charged residue, [17] while Chow et al. also demonstrated a small reduction in viscosity starting at 15 cP to ~8 cP at 130 mg/ml mAb concentration, through charge changes. [15] Larger reductions in viscosity (>4 fold) have been reported by Geoghegan et al. and Yadev et al. using charge change mutations or complementarity-determining region (CDR) swaps. However, this improvement was associated with a large loss of affinity in the former case (12 to 200 fold), [16] and in the latter case, the CDR swaps were done without consideration of the activity of the antibodies, which presumably would not be maintained given the drastic sequence changes. [18] The small data set (4–7 variants) included in each of these studies also makes it difficult to conclude the true potential of generalizing these optimization approaches.

Attempts to generate predictive models of antibody viscosity have also been reported utilizing charge and hydrophobic features. [19–21] Sharma et al. investigated the relationship of FV-charge, -charge asymmetry and -hydrophobicity to the viscosity of a wide range of antibodies. Here the increase in viscosity correlated strongly with the increase in net negative charge and charge asymmetry and, to a lesser extent, increase in the hydrophobic index. [20] Agrawal et al. generated a predictive tool (Spatial-Charge Map (SCM)) which demonstrated the correlation between extent and magnitude of a negative electrostatic patch in the FV domain and the viscosity of several clinical antibodies. [19] Similarly, Tomar et al. trained a support vector machine (SVM) tool to predict the viscosity curve of several antibodies. Here they found that the FV charge played a strong role along with hydrophobicity in predicting the change in viscosity. [21]

Taken together, prior studies have demonstrated that in general, the electrostatics, and to a lesser extent hydrophobicity, play a role in the viscosity of antibodies. Antibodies typically have a pI of 8–9, and therefore in typical formulation buffers of pH 4.5 to 7.5 they have a net positive charge. [22, 23] Additionally, the majority of constant domains have positive net charge at these buffer conditions. [24] At long ranges this would be repulsive, but with non-uniform charge distributions the interaction of negative charge patches can show a general attractiveness to the rest of the antibody and lead to self-association. [18] Concentrations of negative charges in the CDR or variable regions could lead to self-association with other positive patches in the antibody, including the constant region, leading to increased viscosity. [8, 20, 24]

Our work presented here aims to apply these general principles to the optimization of a highly viscous anti-PDGF-BB (platelet derived growth factor B homodimer) antibody while maintaining its binding affinity and overall favorable developability profile. The anti-PDGF-BB antibody had a measured viscosity of 40 cP at 98.8 mg/ml and extrapolated value of 250–300 cP at 150 mg/ml. Our goal was to reduce the viscosity to less than 20 cP at high concentrations (>150 mg/ml). We applied two rounds of structure-based design for the modulation of viscosity while maintaining stability and binding affinity. The resulting optimized lead enables delivery of 150 to 300mg of drug product in single 1 to 2 mL SC injection. In these designs we focused on mutations that would decrease the net negative charge within the variable region, CDRs, and negative charge patches. Sampling a wide space of mutations improved our understanding of parameters governing viscosity vs. other key attributes such as affinity and stability. Our results demonstrate that careful use of structure-based design can avoid the affinity/stability losses reported in previous attempts to remediate viscosity. Viscosity measurements for 40 unique antibody variants were generated with full sequence information reported herein. This wealth of data provides additional resources to the broader scientific community to further our understanding and model building of antibody viscosity and other important attributes in biotherapeutic design.

## Results

### Design of viscosity reducing mutations

**Fig 1:**
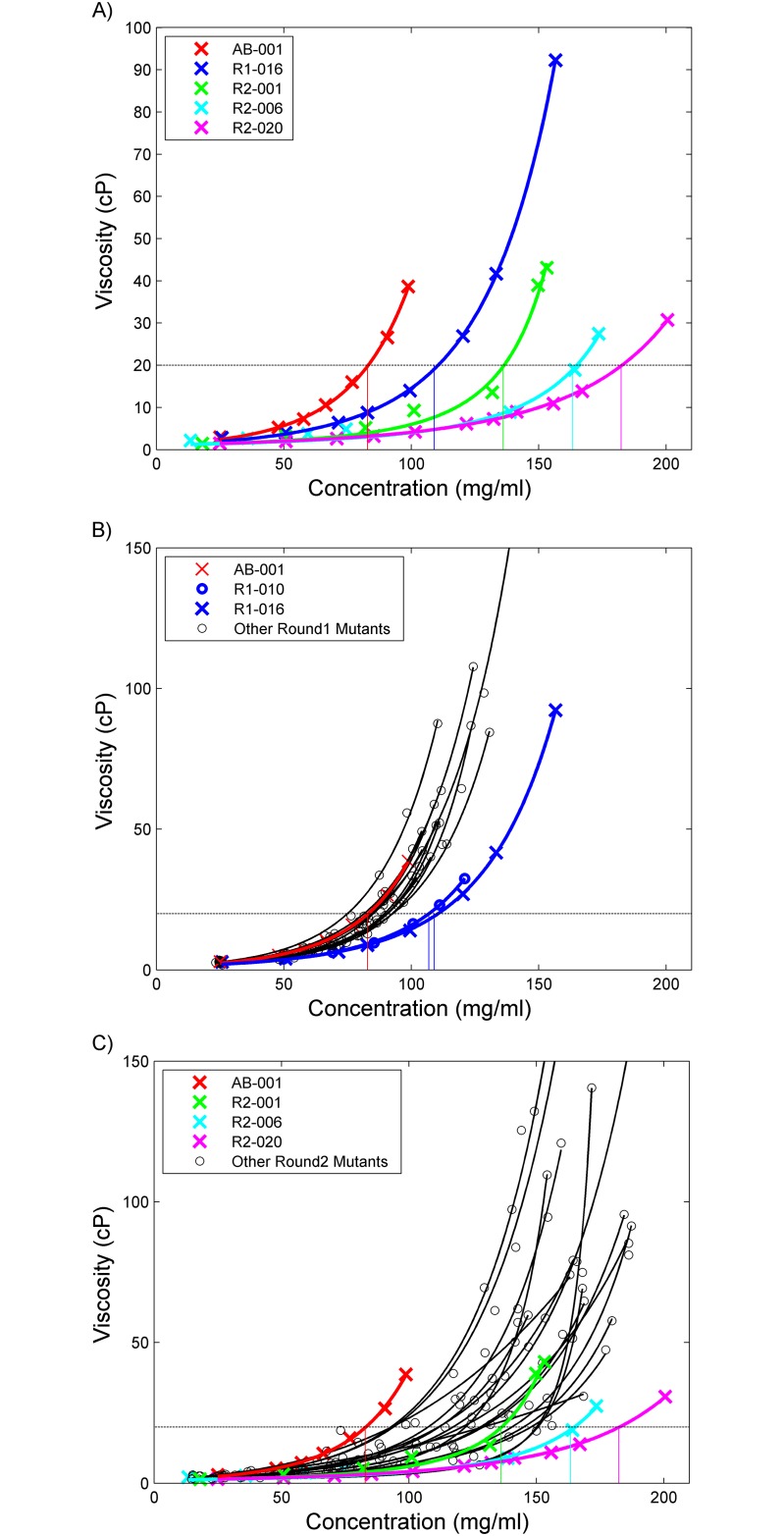
Viscosity analysis of anti-PDGF-BB antibodies. The viscosities of both rounds of antibodies are plotted against their concentrations with exponential curves fit using the Ross and Minton equation. [26] (a) Shows the viscosity for the parental AB-001 (red) along with the round 1 and round 2 mutants that had the biggest decrease in viscosity: R1-016 (blue), R2-001(green), R2-006 (light blue) and R2-020 (magenta). Additionally, vertical lines show the concentration where the viscosity of these antibodies reaches 20 cP. (b) The viscosity data for Round 1 mutants are shown in black, along with the AB-001 data shown in red, and the best clones, R1-010 and R1-016, shown in blue. Additionally, vertical lines show the concentration where the viscosity of these antibodies reaches 20 cP (c) The viscosity data for Round 2 mutants is shown in black, along with R1-001 data shown in red, R2-001 data shown in green, and the best round 2 clones R2-006 in cyan and R2-020 in magenta. Additionally, vertical lines show the concentration where the viscosity of these antibodies crosses 20 cP.

Platelet derived growth factor B (PDGF-B) is a dimeric growth factor whose activation drives proliferation, migration and production of extracellular matrix, and whose signaling has been associated with fibrosis. [25] We had previously developed a potential biotherapeutic anti-PDGF-BB antibody (AB-001) whose binding properties were quite favorable, with affinity to PDGF-BB of 94.8 pM (Table 1). Additionally, most of the biophysical properties such as stability and aggregation propensity of this antibody were acceptable (Table 2). However, the viscosity of AB-001 was significantly higher than desired. As determined by a cone and plate rheometer, the viscosity of AB-001 crosses 20 cP at ~83 mg/ml (Fig 1A). This is well below the desired formulation concentration of >150 mg/ml and would require multiple SC injections to reach this delivery amount. Initial formulation screening of added salt did not show a substantial decrease in viscosity (S1 Fig), leading us to initiate a series of structure-based sequence optimizations aimed at reducing the viscosity to a desirable range (below 20 cP at >150 mg/ml) without significantly impacting activity or other biophysical properties.

**Table 1:** Biacore binding analysis of top viscosity reducing clones.

Ligand	k_a_ (1/Ms)	k_d_(1/s)	K_D_ (pM)
AB-001	5.05E+06±7.82E+05	4.57E-04±6.26E-05	94.83±29.21
R1-016	4.88E+06±4.97E+05	3.57E-04±3.81E-05	74.35±13.74
R2-001	3.09E+06±7.74E+05	2.92E-04±2.79E-05	102.49±32.34
R2-006	3.97E+06±6.25E+05	3.36E-04±6.01E-05	88.80±29.47
R2-020	2.04E+06±1.04E+05	6.10E-04±1.70E-05	298.69±6.83

This table shows the Biacore binding kinetics of AB-001 and the top viscosity reducing clones including the binding on-rate (k_a_), the binding off-rate k_d_ and the equilibrium dissociation constant (K_D_).

**Table 2:** Biophysical properties and off-rate screening of design mutants.

Name	Starting Concentration (mg/ml)	Final High Concentration (mg/ml)	Retention Time (min)	Peak Width	% HMMS	% Main	% LMMS	Tm1 (°C)	Tm2 (°C)	Tm3 (°C)	Apparent Fab Tm (°C)	k_d_ (1/s)
**AB-001**	**4.63**	**98.8**	**9.35**	**0.39**	**0.73**	**99.26**	**0.01**	**69.16 ± 0.09**	**80.89 ± 0.53**	**83.49 ± 0.17**	**82.72**	**3.19E-04**
R1-002	4.94	107.6	9.41	0.43	0.91	99.03	0.06	69.22 ± 0.12	81.73 ± 0.89	83.90 ± 0.25	83.30	3.80E-04
R1-003	3.92	88.9	9.37	0.40	0.61	99.32	0.07	69.34 ± 0.13	82.43 ± 1.00	84.62 ± 0.26	84.21	4.43E-04
R1-004	4.74	104.3	9.37	0.40	0.53	99.39	0.08	69.32 ± 0.13	82.15 ± 1.00	84.44 ± 0.20	83.84	2.58E-04
R1-005	5.25	130.7	9.53	0.43	3.28	96.69	0.04	69.45 ± 0.13	82.21 ± 1.10	84.53 ± 0.21	83.95	3.81E-04
R1-006	5.13	124.3	9.43	0.40	0.59	98.52	0.04	68.11 ± 0.13	82.54 ± 0.75	84.72 ± 0.29	83.81	3.14E-04
R1-007	5.38	156.9	9.34	0.40	0.33	99.04	0.02	68.17 ± 0.12	82.40 ± 0.74	84.41 ± 0.29	83.50	2.82E-04
R1-008	5.19	111.1	9.36	0.39	1.69	98.26	0.05	69.46 ± 0.13	81.64 ± 0.60	84.34 ± 0.15	83.62	5.33E-04
R1-009	4.70	102.2	9.38	0.40	0.59	99.34	0.07	69.42 ± 0.03	79.33 ± 0.47	81.48 ± 0.13	80.95	3.93E-04
**R1-010**	**4.74**	**121.0**	**9.44**	**0.44**	**2.39**	**97.59**	**0.02**	**69.42 ± 0.12**	**82.81 ± 0.73**	**85.63 ± 0.19**	**85.04**	**3.72E-04**
R1-011	4.99	110.4	9.33	0.38	1.35	98.61	0.04	69.37 ± 0.11	81.09 ± 0.67	83.47 ± 0.19	82.60	8.60E-04
R1-012	4.94	109.0	9.35	0.40	2.05	97.79	0.17	68.31 ± 0.12	83.09 ± 0.67	85.30 ± 0.26	84.25	3.04E-04
R1-013	4.70	100.8	9.42	0.44	0.24	99.63	0.13	69.66 ± 0.05	80.17 ± 1.20	82.12 ± 0.18	81.94	2.97E-04
R1-014	4.62	123.5	9.39	0.40	0.33	99.57	0.10	69.28 ± 0.06	80.84 ± 1.50	82.56 ± 0.31	82.20	4.05E-04
R1-015	5.08	104.1	9.35	0.41	0.92	99.07	0.01	67.98 ± 0.11	82.41 ± 1.00	84.22 ± 0.34	83.76	7.52E-04
**R1-016**	**4.93**	**156.6**	**9.41**	**0.41**	**0.65**	**99.23**	**0.12**	**69.38 ± 0.08**	**84.48 ± 0.55**	**86.83 ± 0.24**	**85.51**	**2.89E-04**
R1-017	5.15	89.4	9.4	0.41	0.14	99.79	0.07	69.48 ± 0.09	81.18 ± 1.40	83.07 ± 0.31	82.57	4.71E-04
R1-018	5.17	109.7	9.46	0.44	0.75	99.15	0.10	68.56 ± 0.10	81.15 ± 1.00	83.12 ± 0.28	82.64	4.19E-04
**R2-001**	**5.38**	**153.2**	**10.72**	**0.41**	**0.28**	**99.62**	**0.11**	**69.47 ± 0.11**	**85.16 ± 0.36**	**88.11 ± 0.21**	**86.7**	**4.17E-04**
R2-002	5.46	119.9	10.61	0.41	0.34	99.71	0.00	69.58 ± 0.08	85.21 ± 0.31	88.15 ± 0.16	86.5	4.40E-04
R2-003	5.02	135.6	10.45	0.39	0.60	99.32	0.06	70.21 ± 0.21	74.88 ± 0.24	80.03 ± 0.05	79.8	1.10E-03
R2-004	5.01	164.4	10.64	0.42	0.45	99.43	0.09	69.64 ± 0.03	81.48 ± 1.60	82.92 ± 0.49	82.5	4.39E-04
R2-005	5.08	168.8	10.61	0.42	0.76	99.11	0.11	69.89 ± 0.06	80.17 ± 0.02	80.22 ± 0.09	80.3	3.48E-04
**R2-006**	**5.35**	**173.5**	**10.72**	**0.41**	**0.28**	**99.61**	**0.10**	**69.53 ± 0.09**	**84.20 ± 0.52**	**86.54 ± 0.26**	**85.6**	**3.66E-04**
R2-007	5.00	177.2	10.75	0.44	0.24	99.78	0.00	69.77 ± 0.07	84.13 ± 0.54	86.49 ± 0.26	85.4	4.01E-04
R2-008	5.09	179.7	10.72	0.45	0.47	99.54	0.00	69.58 ± 0.10	84.86 ± 0.44	87.53 ± 0.25	86.1	3.96E-04
R2-009	5.37	168.1	10.69	0.42	0.36	99.59	0.09	69.71 ± 0.06	83.79 ± 0.51	86.05 ± 0.24	85.3	2.65E-04
R2-010	5.00	184.5	10.59	0.40	0.40	99.50	0.10	69.54 ± 0.12	85.01 ± 0.45	87.76 ± 0.27	86.5	weak
R2-011	5.32	168.5	10.45	0.38	0.52	99.42	0.09	69.72 ± 0.04	81.43 ± 1.10	83.09 ± 0.37	82.2	4.14E-04
R2-012	6.79	186.2	11.40	0.58	0.44	99.56	0.00	68.75 ± 0.08	85.17 ± 0.29	88.26 ± 0.14	87.0	3.47E-04
R2-013	5.30	187.3	11.25	0.49	0.33	99.83	0.00	69.66 ± 0.07	85.39 ± 0.27	88.25 ± 0.12	86.6	3.44E-04
R2-014	4.58	187.0	13.01	0.70	2.92	97.08	0.00	68.54 ± 0.11	85.00 ± 0.32	88.22 ± 0.15	87.0	3.96E-04
R2-015	5.03	159.7	11.56	0.54	0.41	99.59	0.00	68.54 ± 0.11	84.23 ± 0.50	86.83 ± 0.27	85.7	8.34E-04
R2-016	6.47	146.7	11.44	0.52	0.20	99.80	0.00	68.61 ± 0.12	82.45 ± 0.64	84.99 ± 0.19	84.4	2.66E-04
R2-017	5.21	154.2	11.53	0.53	0.17	99.83	0.00	68.55 ± 0.08	79.42 ± 0.01	84.66 ± 0.15	79.3	6.73E-04
R2-018	5.10	171.7	10.84	0.43	0.33	99.66	0.00	69.59 ± 0.07	84.54 ± 0.46	87.10 ± 0.23	85.8	3.86E-04
R2-019	5.20	163.2	10.44	0.59	1.45	98.59	0.00	69.47 ± 0.07	82.53 ± 0.64	84.56 ± 0.31	83.4	6.73E-04
**R2-020**	**12.69**	**200.5**	**12.06**	**0.59**	**0.15**	**99.85**	**0.00**	**68.65 ± 0.11**	**85.57 ± 0.21**	**90.00 ± 0.10**	**89.2**	**3.86E-04**
R2-021	8.23	174.0	11.40	0.51	0.30	99.71	0.00	68.05 ± 0.14	84.69 ± 0.50	87.33 ± 0.29	86.1	weak
R2-022	6.94	160.8	11.44	0.53	0.55	99.45	0.00	68.55 ± 0.14	84.97 ± 0.46	87.78 ± 0.25	86.2	3.61E-04

This table includes the biophysical properties of AB-001 and two rounds of design mutants. This includes the initial concentration and final high concentration for viscosity measurements, analytical size exclusion chromatography (aSEC) retention time, peak width and species fractions (main, high molecular mass species (HMMS), low molecular mass species (LMMS)), and differential scanning calorimetry (DSC) thermal stability properties. Additionally, the off-rate (k_d_) screening measurements are also presented where R2-010 and R2-021 showed “weak” binding and the curve was not fit.

**Fig 2:**
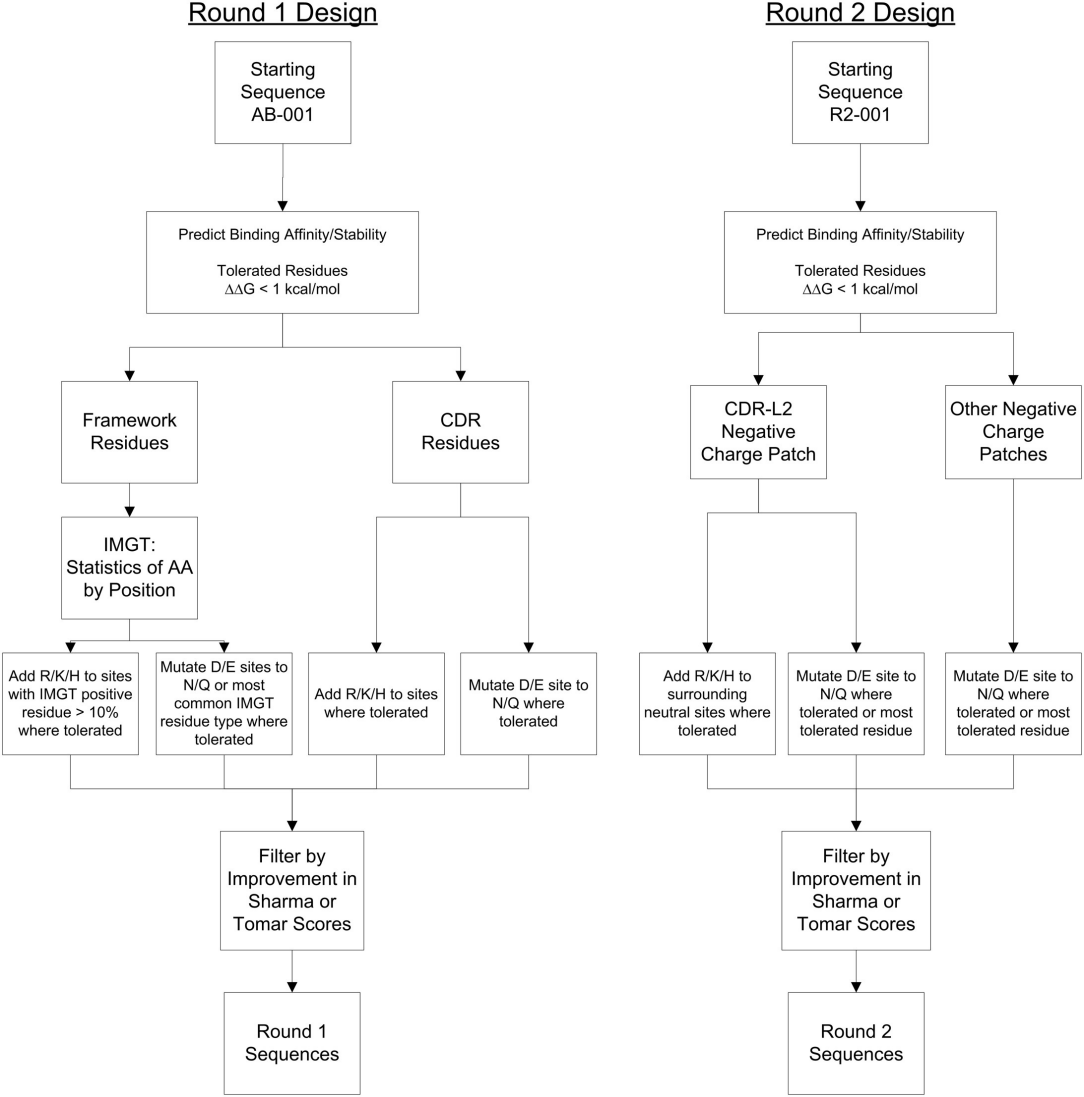
Flowchart for the round 1 and round 2 design strategy.

**Fig 3:**
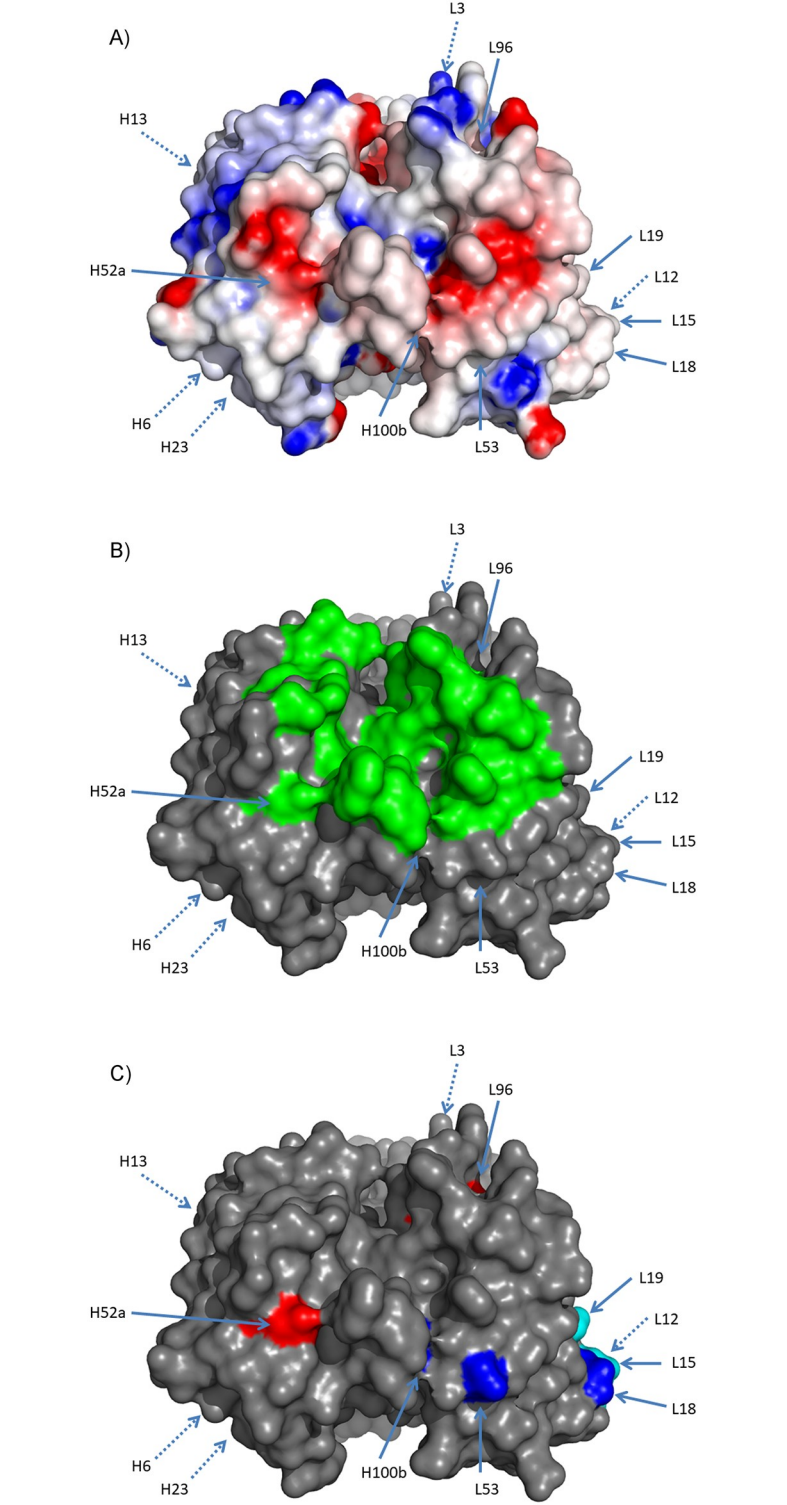
Round 1 design of viscosity reducing mutations. Mutations in round 1 were designed to reduce the overall negative charge and to reduce the negative charge patches. These patches were identified by generating an electrostatic potential energy surface using Delphi [30–32] seen in (a). Here red is negative charge potential and blue is positive charge potential. Mutations were also generated to avoid the interface residues (green) that are identified from the PDB structure 4qci and seen in (b). The mutants that were selected are shown in (c) where removal of negative charge sites is in red, addition of positive charge sites is in blue and the mutants for changing the FW1 to remove the clipped species are shown in light blue. All sites are annotated with Kabat positions.

In previous work we solved the crystal structure of a AB-001 variant bound to PDGF-BB at a resolution of 2.3 Å (PDB id: 4QCI). [27] The variable region of this antibody was 97% identical to AB-001, containing only 5 framework differences in the light chain (LC), and 2 framework differences in the heavy chain (HC) (S1 Table). This allowed us to generate a high-quality homology model of AB-001 in complex with PDGF-BB to get a detailed understanding of not only the interface but also the surface properties of the antibody. The initial design to identify viscosity reducing mutations (Round 1) was focused on mutations that modified the charge pattern of AB-001, particularly reducing the number of negative charges, or negative charge patches. These types of charge changes were identified by previous work as playing an important role in the viscosity. [18, 28, 29] Additional design constraints were implemented in order to maintaining the binding affinity and other important developability parameters in the molecule including stability, solubility, and immunogenicity. To address this, we applied the following design strategy (Round 1 in Fig 2) to identify mutations that would introduce a net positive charge change (addition of positive charge residue or removal of negative charge residue), be predicted to maintain affinity and stability, and limited to sites in the CDRs or mutations in the framework that are present in common human sequences (S2 Table). The detailed steps of this method are **(1)**: For each site, the change in stability upon mutation and the change in binding affinity upon mutation was calculated using Discovery Studio 3.5 with the AB-001:PDGF-BB complex. From these calculations, a set of mutations were determined that were predicted to increase the net charge while not affecting stability or binding affinity (tolerated mutations predicted to have ΔΔG < 1.0 kcal/mol). **(2)** Glu and Asp sites in the CDR regions were further limited to mutations to Gln or Asn, respectively, where tolerated. **(3)** Neutral sites in the CDR regions were allowed to be mutated to Arg, Lys or His if tolerated. **(4)** The framework mutations were then limited to those that had a high probability (>10%) of a positive residue (Lys or Arg) in human antibody sequences or sites with a wild-type Glu or Asp that had a high probability (>10%) of another neutral or positive residue. **(5)** For all possible mutations that met these criteria, we then characterized the mutations using two different published methods for the prediction of viscosity. One was a sequence based method described in Sharma et al. (referred to as the Sharma method) which predicts the viscosity from sequence based upon Fv charges, VH/VL charge similarities and hydrophobic index. [20] The second was a similar method (Tomar method) that uses the structure to calculate the VH, VL and hinge charges along with the hydrophobic surface area to predict the concentration dependent viscosity. [21] All allowed mutations had a reduced value for either the Tomar or Sharma methods. **(6)** From here a prioritized set of designs was selected which included mutations that were in close proximity to the negative charge patch in the CDR, as determined by calculation of a electrostatic surface potential map using the Poisson Boltzmann calculator DelPhi [30–32], and framework mutations including one double mutant whose total segment or surrounding residues were found in human germlines. All VH mutants also included a K94R mutant to remove any potential glycation risk. **(7)** Finally, combinations of heavy chain and light chain mutations were also selected. These were done because analysis of previously reported data comparing viscosity to charge [8, 20, 21, 24, 29] suggests that it may require a charge change of two or more to reduce the viscosity to desirable levels. This set of 17 Round 1 mutants (R1-002 to R1-018) is shown in Fig 3 and Table 3, with sequences in S1 Table.

**Table 3:** In silico and viscosity properties of design mutants.

Name	Heavy Chain	Light Chain	Fv Charge	Sharma Score	Tomar Score	SCM	Viscosity (cP) @ 100 mg/ml	Viscosity (cP) @ 150 mg/ml	Conc. (mg/ml) @ Visc. of 20 cP
**AB-001**	**AB-001_HC**	**AB-001_LC**	-2.01	78.17	0.759	-2213	40.4 ± 1.1*	439.7 ± 127.8*	82.9 ± 2.5
R1-002	E6Q_K94R	AB-001_LC	-1.07	76.87	0.778	-2008	29.3 ± 0.9	288.3 ± 91.0*	90.1 ± 3.5
R1-003	Q13K_K94R	AB-001_LC	-1.01	74.96	0.769	-1985	39.5 ± 2.5*	522.9 ± 255.9*	84.9 ± 4.7
R1-004	A23R_K94R	AB-001_LC	-1.01	66.55	0.788	-1961	36.0 ± 1.1	310.4 ± 94.4*	85.1 ± 3.2
R1-005	D52aN_K94R	AB-001_LC	-1.04	77.92	0.73	-1838	26.0 ± 1.5	190.2 ± 17.4*	95.9 ± 3.0
R1-006	Q100bR_K94R	AB-001_LC	-1.01	71.16	0.653	-1941	40.8 ± 1.1	313.7 ± 21.6*	81.7 ± 2.0
R1-007	E6Q_Q13K_K94R	AB-001_LC	-0.07	77.86	0.777	-1988	35.9 ± 5.4	233.2 ± 10.3	89.6 ± 3.7
R1-008	AB-001_HC	E3V	-1.07	73.93	0.529	-2085	30.7 ± 1.0	567.2 ± 181.1*	90.8 ± 3.0
R1-009	AB-001_HC	T18R	-1.01	59.05	0.507	-2180	29.8 ± 0.8	430.4 ± 162.3*	90.8 ± 5.1
**R1-010**	**AB-001_HC**	**N53K**	-1.01	64.64	0.602	-1898	15.9 ± 0.5	98.6 ± 11.4*	106.9 ± 5.1
R1-011	AB-001_HC	D96N	-1.04	67.85	0.607	-2035	56.8 ± 2.5	519.4 ± 164.6*	77.0 ± 2.2
R1-012	E6Q_Q13K_K94R	E3V	0.87	67.48	0.639	-1861	39.2 ± 1.5	471.0 ± 145.0*	82.5 ± 3.5
R1-013	E6Q_K94R	T18R	-0.07	55.84	0.614	-1972	29.7 ± 1.6	414.3 ± 285.4*	90.9 ± 7.3
R1-014	E6Q_Q13K_K94R	T18R	0.93	54.13	0.599	-1983	27.5 ± 2.4	414.7 ± 116.6*	89.2 ± 4.2
R1-015	E6Q_Q13K_K94R	D96N	0.9	61.92	0.602	-1817	41.3 ± 1.8	452.1 ± 211.4*	82.6 ± 2.4
**R1-016**	**E6Q_Q13K**_K94R	**N53K**	0.93	58.99	0.645	-1706	14.5 ± 0.3	73.1 ± 0.6	109.0 ± 4.8
R1-017	Q13K_K94R	T18R	-0.01	54.41	0.594	-1949	42.6 ± 2.8*	1534 ± 1077*	85.7 ± 4.5
R1-018	Q100bR_K94R	T18R	-0.01	51.83	0.559	-1914	33.2 ± 0.6	415.8 ± 70.6*	87.6 ± 2.7
**R2-001**	**R2-001_HC**	**R2-001_LC**	3.87	28.09	0.221	-1503	6.0 ± 2.0	37.3 ± 3.3	132.1 ± 7.2
R2-004	R2-001_HC	Y49H	4.63	22.71	0.099	-1480	14.8 ± 1.3	54.4 ± 2.0	110.8 ± 5.7
R2-005	R2-001_HC	Y49R	4.87	19.43	0.078	-1456	9.7 ± 5.9	36.7 ± 10.8	107.2 ± 17.7
**R2-006**	**R2-001_HC**	**S52K**	4.87	20.74	0.072	-1470	4.0 ± 1.2	12.6 ± 2.6	163.4 ± 21.7
R2-007	R2-001_HC	S65K	4.87	20.74	0.054	-1433	6.4 ± 3.0	21.1 ± 6.5	134.6 ± 17.5
R2-008	R2-001_HC	S67K	4.87	20.74	0.062	-1460	6.5 ± 2.7	23.0 ± 6.8	148.6 ± 16.5
R2-009	R2-001_HC	G68K	4.87	20.18	0.076	-1469	3.6 ± 0.7	19.3 ± 3.7	141.6 ± 10.6
R2-010	Y97H	R2-001_LC	4.63	24.61	0.246	-1656	9.6 ± 0.7	35.1 ± 1.6	122.6 ± 11.7
R2-011	Q100bK	R2-001_LC	4.87	23.79	0.209	-1500	13.1 ± 2.2	25.8 ± 2.4	131.3 ± 11.8
R2-012	D52aK	R2-001_LC	5.85	20.9	0.226	-1273	12.3 ± 3.3	38.9 ± 6.0	133.1 ± 5.7
R2-013	D52aN	R2-001_LC	4.85	24.77	0.244	-1357	7.1 ± 1.4	26.4 ± 4.3	133.2 ± 21.6
R2-014	D53Q	R2-001_LC	4.85	24.68	0.17	-1378	12.5 ± 0.8	50.8 ± 2.1	121.4 ± 5.0
R2-015	D61N	R2-001_LC	4.85	24.77	0.237	-1446	15.8 ± 1.9	83.2 ± 3.2	107.3 ± 6.0
R2-016	D101N	R2-001_LC	4.85	24.77	0.231	-1416	14.1 ± 1.2	66.5 ± 3.0*	111.2 ± 5.0
R2-017	D101Y	R2-001_LC	4.85	26	0.239	-1564	8.8 ± 0.8	83.5 ± 2.8	115.4 ± 5.4
R2-018	R2-001_HC	D26N	4.85	22.34	0.06	-1346	3.4 ± 0.9	19.9 ± 7.1	127.4 ± 24.2
R2-019	R2-001_HC	D50L	4.85	24.22	0.079	-1503	23.2 ± 6.2	59.5 ± 6.6	91.3 ± 15.0
**R2-020**	**R2-001_HC**	**D51N**	4.85	22.34	0.05	-1363	4.1 ± 0.1	10.0 ± 0.2	182.4 ± 2.0
R2-021	R2-001_HC	D96K	5.85	16.96	-0.104	-1197	24.2 ± 4.8	118.9 ± 10.9	100.2 ± 3.5
R2-022	R2-001_HC	D96N	4.85	22.34	0.072	-1311	25.2 ± 1.6	134.7 ± 2.6	98.5 ± 2.5

This table includes the identity of mutations, charge and in silico viscosity predictions from the Sharma, Tomar and SCM methods, the viscosities at 100 mg/ml, 150 mg/ml and the concentration at which the viscosity is 20 cP for the parental AB-001 and two rounds of design mutants. All sites are annotated with Kabat positions. Viscosities with (*) are extrapolated from the data fitting as the highest concentration obtained for this antibody was below 100mg/ml or 150 mg/ml respectively.

### Biophysical characterization

The aggregation state for AB-001 and the round 1 designed antibodies was evaluated using analytical size exclusion chromatography (aSEC). A modified mobile phase containing arginine in PBS was used for both parental and all variants to minimize protein column matrix interaction. Antibody samples at various concentrations were analyzed to confirm all viscosity mutants contained <5% aggregation with good protein mass recovery from aSEC column (Table 2). Differential scanning calorimetry (DSC) was performed to determine the thermal stability of the viscosity mutants monitored by heat capacity change as function of temperature where T_m_ is defined as the midpoint of unfolding transition. Comparison to that of the parental molecule AB-001 was used to ensure mutations did not adversely change the overall stability of these mutants. All mutants are considered stable with the first unfolding transition, Tm1, over 65°C and had comparable DSC profiles (Table 2). Clones R1-010 and R1-016 showed higher apparent Fab thermal stability compared to the parental clone.

### Viscosity measurements

Viscosity curves were generated for the parental AB-001 and round 1 designed mutants using an Anton Paar rheometer. The viscosity data (S3 Table) was plotted against the respective range of protein concentrations (mg/ml) to generate the viscosity curves for each mutant (Fig 1b). Except for clones R1-007 and R1-016, many of the clones concentrated poorly in the filtration units with most of them unable to achieve concentrations >100 mg/ml and attempts to do so resulted in significant loss of protein. The final concentrations for the designed viscosity mutants are listed in Table 2. The viscosity data was fit to the exponential Ross-Minton equation [26] (see Methods, Eqs 2 and 3) to interpolate/extrapolate the viscosities at 100 mg/ml, 150 mg/ml and the concentration where the viscosity reaches 20 cP (Table 3). The data for all 18 antibodies fit well to this equation with correlation coefficients (R^2^) of > 0.99 (S2 Fig). The viscosity profiles for most of the clones were similar to that of the parental AB-001, with the exception of clones R1-016 and R1-010 which were the only clones that had a 3-fold decrease in viscosity at 150 mg/ml and a 20% increase in concentration at which the viscosity reached 20 cP. These two clones had a higher protein concentration of ~109 and 107 mg/ml at the maximal viscosity threshold of 20 cP, and at 150 mg/ml had reduced viscosity to approximately 73 and 99 cP (interpolation for R1-016 and extrapolation for R1-010).

### Binding analysis

Biacore off-rate analysis was performed on the parental antibody along with the 17 round 1 design variants. The off-rate of all 18 clones was very similar with no significant increase (Table 2). Clone R1-016 showed the most significant change in viscosity, therefore we carried out a full kinetics analysis to compare with the parental for binding affinity. The average binding affinity of clone R1-016 was 74.4 pM which was comparable to the parental value of 94.8 pM (Table 1). The introduction of these mutations did not significantly change the binding affinity.

### Further viscosity optimization

The data from the first round of mutants shows that only 2 out of the 17 designs exhibited significant change in their viscosities. These were clone R1-010, which has the light chain mutation N53K, and clone R1-016, which has the same light chain mutation N53K, along with two heavy chain mutations, E6Q and Q13K. The only significant outlier of the remaining mutants was clone R1-011 (LC mutation D96N) which had slightly increased viscosity over parental. All of the mutations had a net positive charge change and were predicted by the Sharma Score and Tomar Score to have reduced viscosity. Given that the location of the mutation seems to play a significant role in the reduction of viscosity a second round of mutations were designed to increase the charge in a manner similar to that of the N53K mutation.

**Fig 4:**
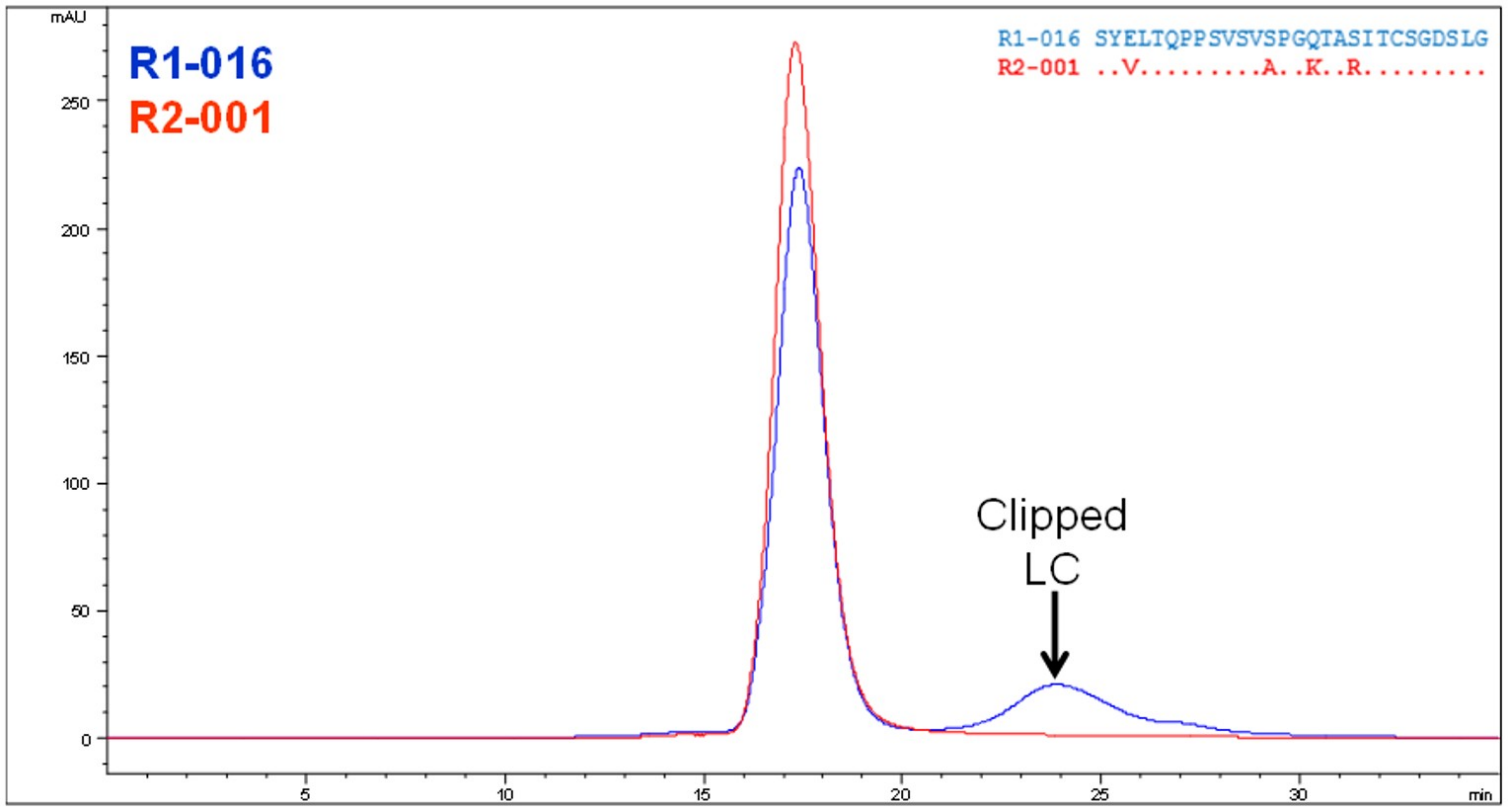
Removal of a low-molecular weight clipping species. aSEC analysis shows that a clipped species is formed during production of the R1-016 (blue). MS identified that being a 3 residue N-terminal truncation of the LC. Mutation of the FW1 region to the FW of IGLV3-21*01, clone R2-001, removes this clipped species (red).

Design of the second round of mutations was to be based upon R1-016 which showed the largest improvement in viscosity with no change in binding affinity. However, a larger scale expression and purification of R1-016 (and other first round clones) revealed a significant amount of low-molecular weight species (~10%) (Fig 4) that was confirmed by mass spec to be clipping of the light chain after position Glu3. This was unexpected and represented a significant complication for downstream purification process development. To mitigate this clipping the FW1 was changed to that of a different lambda germline, IGLV3-21*01. This clone (R2-001) mutated the Glu at position 3 along with 3 additional mutations (S13A, Q16K and S19R). These framework mutations would result in net positive charge change of plus 3 and reduction in the Tomar and Sharma scores, in addition to, the LC mutation S19R was in close proximity to the key N53K mutation that reduced viscosity in the first round (Fig 3). Therefore, this new framework variant was expected to maintain or possibly further reduce the viscosity of the antibody.

This new clone (R2-001) was expressed, purified and characterized as before. This new design was successful in completely removing the clipping of the R1 clone series (Fig 4), while maintaining other biophysical properties (Table 2) and binding affinity (Table 1). Additionally, the viscosity was measured using the same method as in round 1 and showed a further improvement over clone R1-016 (Fig 1a) reducing the viscosity at 150 mg/ml to approximately 38 cP and reaching a higher concentration of 132 mg/ml at 20 cP (Table 3).

**Fig 5:**
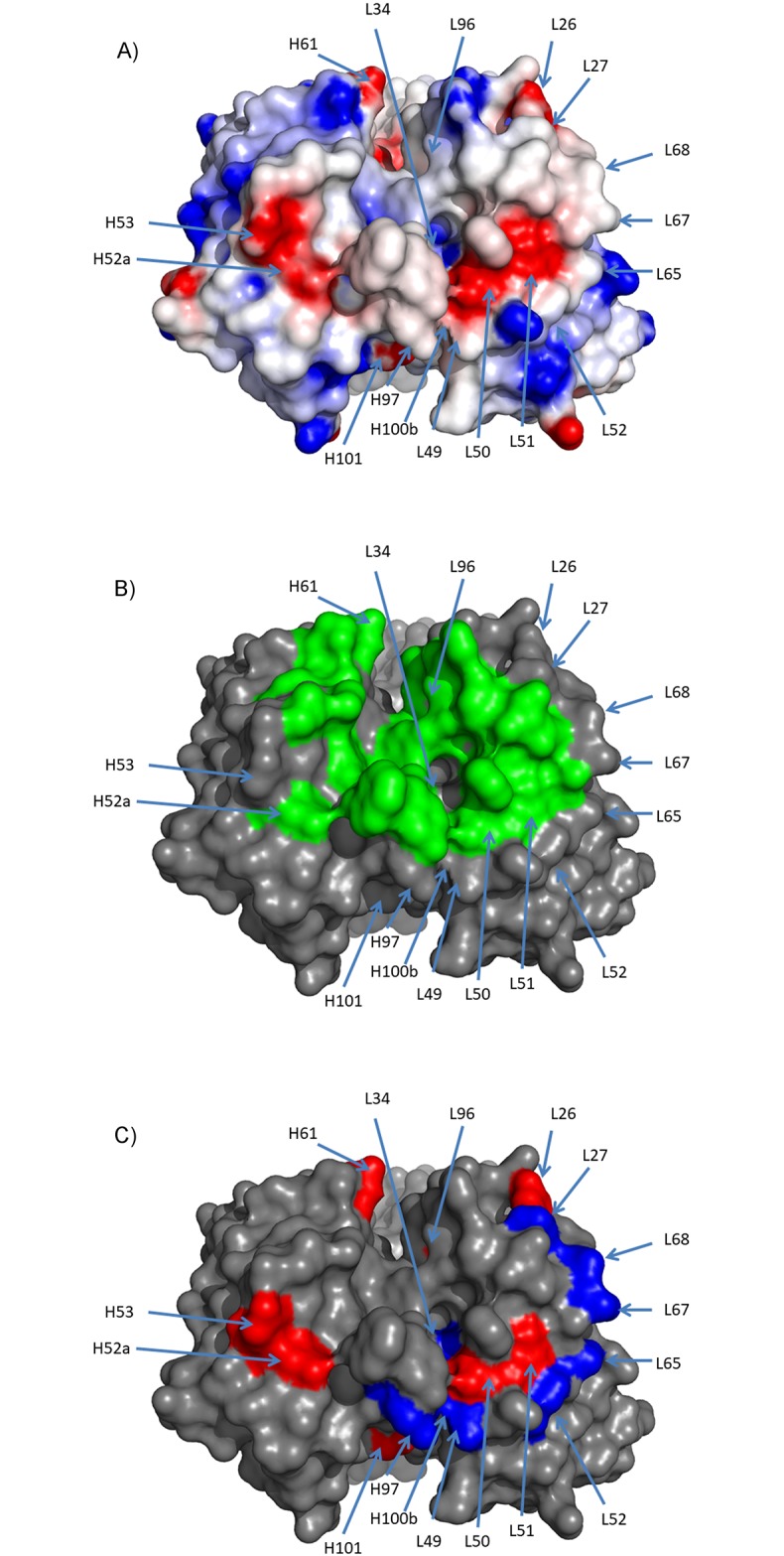
Round 2 design of viscosity reducing mutations. Mutations in round 2 were based off of R2-001 and were designed to be similar to the L53 mutation, introducing net-positive charge changes adjacent to the proximal negative charge patch. These mutations were designed to not interfere with binding. Additionally, negatively charged residues in negative charge patches were also designed to be removed. Here the mutations were selected to minimize the effect on binding. The charge patches were identified by generating an electrostatic potential energy surface using Delphi [30–32] seen in (A). Here red is negative charge potential and blue is positive charge potential. The interface residues (green) that are identified from the PDB structure 4qci are seen in (b). The mutants that were selected are shown in (c) where removal of negative charge sites is in red and addition of positive charge sites is in blue. All sites are annotated with Kabat positions.

Based upon the location of the viscosity reducing LC mutation N53K and the FW change mutations, a new set of clones (Round 2) were designed to further reduce viscosity using the method shown in Fig 2. **(1)** Similar to the first round of designs, all potential mutations were computationally screened for binding affinity and stability (S2 Table). **(2)** A set of positive charge introduction mutations were generated around the CDR-L2 negative charge patch near the N53K mutation. **(3)** In addition, given the possible importance of the charge patch contributing to viscosity, additional negative charge removal mutations were also introduced. This included the negative charge patch in the LC (containing L50 and L51 sites) along with an additional negative charge patch in the HC chain (containing H52a and H53 sites) (see Fig 5a). Unlike the positive charge change mutations, some of these were part of the binding interface and were predicted to have a small effect on the binding affinity (predicted ΔΔG of 1 to 2 kcal/mol, S2 Table). This set of 21 second round of mutations (R2-002 to R2-022) and the reference R2-001 are shown in Table 3 and Fig 5, with sequences in S1 Table. These 22 mutants were expressed and purified, and biophysical characterizations were performed the same as for round 1. All clones showed less than 5% high molecular mass species (HMMS), and all maintained acceptable thermal stability with Tm1 > 65°C (Table 2). Mutants R2-002 and R2-003 both formed precipitates during concentrating and were discarded.

Viscosity curves were generated for round 2 design mutants in the same manner as the round 1 design (Fig 1c) with the data in S3 Table. These curves were fit well using the Ross-Minton equation as described above (with correlation coefficients (R^2^) of > 0.96) and the viscosity at 100 mg/ml, 150 mg/ml and the concentration which the viscosity reaches 20 cP are shown in Table 3. Two of the mutants showed substantial reduction in viscosity, having 3-fold decrease in viscosity at 150 mg/ml and a 20% increase in concentration at which the viscosity reached 20 cP. These were R2-006 which has a LC mutation S52K and R2-020 which has a light chain mutation D51N. These mutations improved the viscosity profile and increased the concentration at which they reached the maximal viscosity threshold of 20 cP; increasing from ~132 mg/ml for R2-001 to ~163 mg/ml for R2-006 and ~182 mg/ml for clone R2-020. All other mutants had similar or increased viscosities to the starting clone R2-001 (Fig 1c). As compared to the parental clone AB-001 which had extrapolated viscosity of >450 cP at 150 mg/ml, clones R2-006 and R2-020 had viscosities of 12.6 cP and 10.0 cP at the same concentration of 150 mg/ml, respectively (Fig 1a and Table 3).

Antibodies from the second round of mutations were screened for off-rate similarly to those clones seen in the first round of designs (Table 2). Clones R2-011 and R2-021 showed weak binding, and clones R2-004, R2-019 and R2-022 showed low R_Max_ values. The remaining clones had similar off-rates to the parental clone. Additional kinetic analysis was performed for those viscosity reduced clones R2-001, R2-006 and R2-020 (Table 1). R2-001 (K_D_ = 88.8 pM) and R2-006 (K_D_ = 102.9 pM) had similar binding affinities to the Parental AB-001 (K_D_ = 94.8 pM). R2-020 (K_D_ = 298.7 pM) had a ~3X reduction in binding affinity as compared to the other clones. This was in-line with what was predicted using the computational affinity prediction methods as the D51N mutation of R2-020 was predicted to play a significant role in the binding interface.

### Non-specificity analysis

The round 1 and round 2 viscosity reducing mutants all had a positive net charge change ranging from +3 for R1-016, +6 for R2-001 and +7 for R2-006 and R2-020. Excess positive charge has been associated with poor biophysical properties, non-specificity and poor clearance. [20, 33, 34] Sharma et al. demonstrate that the optimal Fv charge of 0 to 6 is correlated with reduced risk of poor clearance properties. [20] The parental AB-001 and R1-016 had low Fv charge of -2.01 whereas the optimized mutants all had Fv charge in the optimal range with 0.93 for R2-016, 3.87 for R2-001, 4.87 for R2-006 and 4.85 for R2-020 (Table 4). Rabia et al. analyzed 137 clinical antibodies and demonstrated a correlation of a predicted net positive CDR charge at pH 7.4 with poor biophysical properties including non-specificity and self-association. [33] They showed that a net CDR charge > 0 showed increased number of red flags in a range of non-specificity assays. All of this set of mutants has <0 net CDR charge. The parental AB-001 has a net CDR charge of -3.7, while R1-016 is -2.7, R2-001 is -2.7, R2-006 is -1.7 and R2-020 is -1.7 (Table 4).

**Table 4:** FV-charge, CDR-charge and non-specificity properties of top viscosity reducing clones.

Ligand	Fv Charge	CDR Charge	DNA Score	Insulin Score	AC-SINS Score
AB-001	-2.01	-3.7	2	2	22
R1-016	0.93	-2.7	2	4	24
R2-001	3.87	-2.7	2	6	24
R2-006	4.87	-1.7	2	6	25
R2-020	4.85	-1.7	3	5	4

The Fv Charge was calculated using the method described in Sharma et al. [20] The CDR Charge was calculated using the method in Rabia et al. [33].

To further validate the effect of the addition of these positive charges we performed two non-specificity assays (DNA binding ELISA and Insulin Binding ELISA) along with a self-association assay (AC-SINS) on the top round 1 and round 2 charge mutants (Table 4). High values (> = 11) in multiples of these assays has been showed previously to correlate with poor clearance in PK studies. [35] None of the antibodies had high values in the DNA and Insulin assays. AB-001 has a high value in the self-association AC-SINS assays. None of the other mutants show an increase in AC-SINS score, with R2-020 showing a significant decrease. These assays suggest that the overall non-specificity of these mutants does not increase, or, in the case of R2-020, may decrease.

### Analysis of viscosity reducing mutations

**Fig 6:**
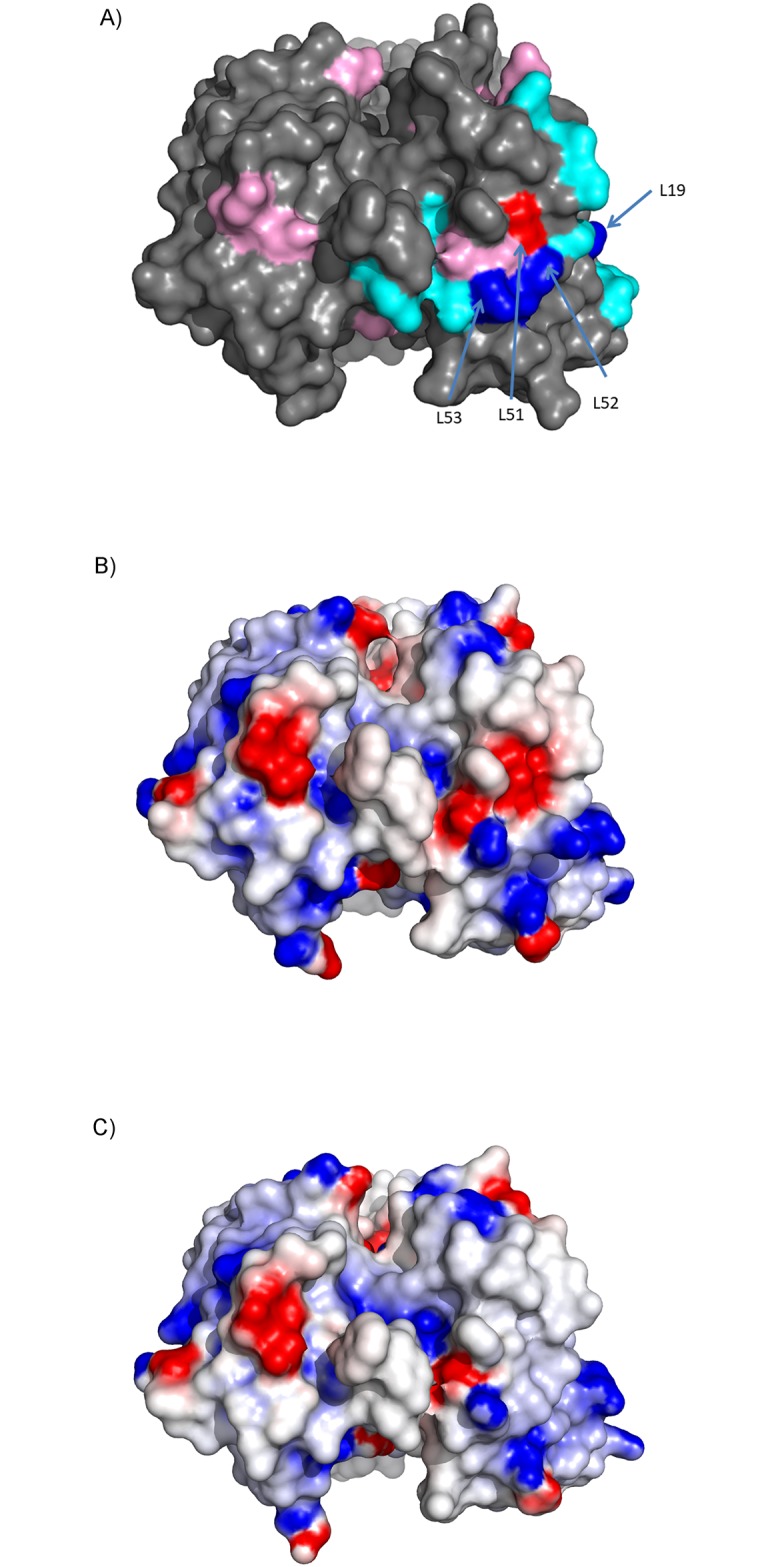
Analysis of the effect of mutations on viscosity. Two sets of mutations were generated and only a few showed an effect at decreasing the overall viscosity of the antibody. In (A), residues that added a positive charge and reduced viscosity are shown in blue, and those that had no effect or increased the viscosity are shown in light blue. Residues that removed a negative charge and reduced the viscosity are shown in red and those that had no effect or increased the viscosity are shown in magenta. The 4 sites that had the largest effect are all clustered together. All sites are annotated with Kabat positions. As comparison to the parental AB-001 and round 2 parental R2-001, (B) shows the electrostatic potential energy surface of clone R2-006 which had reduced viscosity with maintained activity and (C) shows the electrostatic potential energy surface of R2-020 which has reduced viscosity with 3X loss of activity.

The two rounds of designs were based upon the hypothesis that reduction in negative charge and/or reduction in negative charge patches would result in lower viscosity variants. In these designs the most significant changes in viscosity (3 fold decrease in viscosity at 150 mg/ml and 20% increase in concentration with viscosity at 20 cP) were associated with LC mutations N53K, S52K, D51N and FW1 changes to the IGLV3-21*01 germline, including S19R which is in close spatial proximity to LC positions 53, 52 and 51. A number of other mutants had similar changes in charge yet none of the HC mutations (E6Q, Q13K, A23R, D52aN, Q105R, D52aK, D53Q, D61N, Y97H, Q100bK, D101N, D101Y), or other LC mutations (E3V, T18R, D96N, S27K, H34K, Y49H, Y49R, S65K, S67K, G68K and D96K) showed a significant change in the viscosity. All of the successful viscosity reducing mutations had net positive charge changes, but there were many similar charge changes that had no effect or negative effect on viscosity. Most of the mutations that were effective were centered on the negative charge patch in the LC but mutations that had the largest influence were localized to a small portion of this near the middle of CDR-L2 (Fig 6a). The proximity and effect that these mutations had on this negative charge patch can be seen in the difference between the electrostatic surface of the parental clone (Fig 3a) and the optimized clones R2-006 (Fig 6b) and R2-020 (Fig 6c).

**Fig 7:**
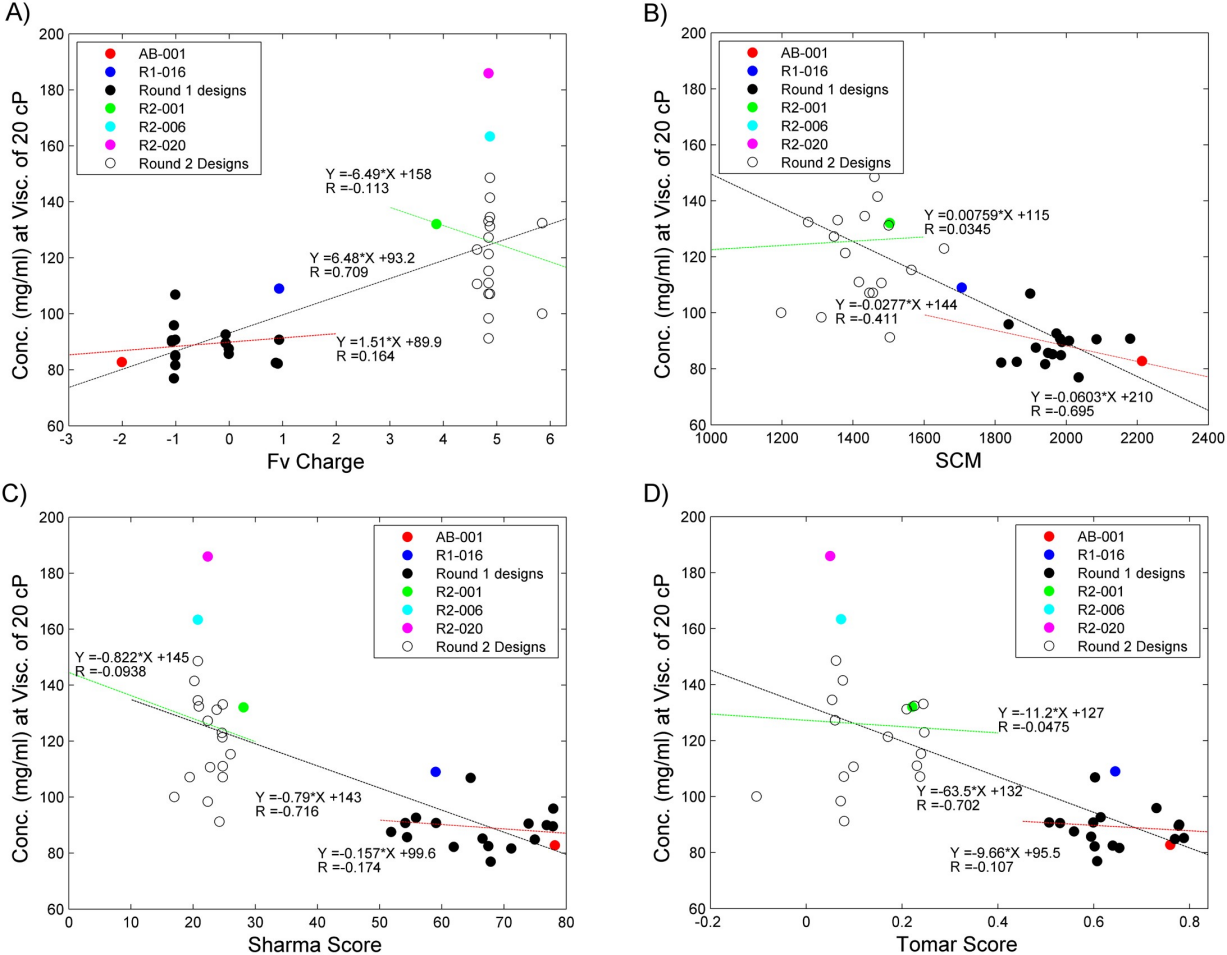
Analysis of charge-based prediction of viscosity. The concentrations where the viscosity reaches 20 cP of the round 1 and round 2 mutants are plotted against the (a) Fv-Charge, (b) SCM method, (c) Sharma Score and (d) Tomar Score. For each of these AB-001 is shown in red, R1-016 is in blue, the remaining round 1 designs in filled black circles, R2-001 in green, R2-006 in cyan, R2-020 in magenta and the remaining Round 2 designs are in empty black circles. The linear fit lines are shown on the charts along with the correlation value. Round 1 linear fit is shown as a red dashed line, Round 2 linear fit as a green dashed line and the overall linear fit is shown as a black dashed line.

Of the designs that were made, only 2 from the first round and 2 from the second round produced a substantial reduction in viscosity. The designs from the first round and second round had a similar reduction in the net negative charge and predicted change by the Sharma and Tomar scoring methods, which had been shown previously to be associated with a decrease in viscosity. [20, 21] Therefore, it was somewhat surprising that the success rate was so low. Because of this we further examined how viscosity correlated with the change in charge, Fv-charge, and the change in the surface charge patches, as predicted by SCM, comparing those to the two prediction methods utilized in the design, Sharma score and Tomar Score (Table 3). SCM is a method which uses the antibody structure to identify the asymmetry of surface charges and charge clusters. [19] When comparing Fv-Charge or SCM score against the concentration where the antibody’s viscosity is 20 cP, the overall correlation coefficients (R) were good at 0.71 and -0.70 respectively, which is similar to the predictive performance of the Sharma Score (R = 0.72) and the Tomar Score (R = 0.70) (Fig 7). However, a closer inspection showed that much of the significant correlation for these methods is associated with the ability to distinguish the first and the second-round mutants. R2-001, which had a significant reduction in viscosity, also had a +4 charge change, and reduced SCM, Tomar and Sharma Scores (as compared to AB-001), was the center for the design for all of the round 2 variants, thus skewing the overall composition of the dataset.

As an additional test we determined the ability of these methods to differentiate the individual mutants within the first round and second rounds of designs. When looking at the correlation of the first round of designs, the correlation (R) of the Fv-charge decreases to 0.16, SCM to -0.41, Sharma Score to 0.17 and Tomar Score to 0.10. Despite the poor correlation, the SCM method does identify the top viscosity reducing clones (R1-016) and has the second best (R1-010) in the top 5. This is likely due to the fact that the SCM score can identify reduction in structurally concentrated charge areas which correlated with the reduction in viscosity, whereas FV-charge, Sharma Score and Tomar Score all view charge mutations similarly regardless of localization within the VH or VL. The same analysis was done on the second round of mutations where the correlation coefficient R was -0.13 for the Fv-charge, 0.03 for the SCM method, 0.09 for the Sharma Score, and 0.05 for the Tomar score. Here all methods have weak correlation and are not able to separate the two mutants with significantly reduced viscosity. Despite the fact that a stronger correlation of these type of predictions has been shown to work across sets of antibodies that are quite different, [19–21] in this case they do not perform as well on this set which consists of sequence related antibody variants with similar changes in charge properties.

## Discussion

The ability to deliver antibodies at high concentration may be required to achieve efficacious doses or practical dosing intervals. Viscosity limits the ability to concentrate many antibodies. [8, 20, 21, 24, 29] Mitigation of high viscosity has been difficult, as in many cases the residues that are responsible for the high viscosity may be located within the paratope of the antibody. [15–18] This becomes particularly challenging for those antibodies with high binding affinity. Here we used structure guided design to introduce sequence variants and reduce the viscosity of the high affinity, but highly viscous, anti-PDGF-BB antibody AB-001. The concentration where antibody variant R2-006 reached the 20 cP maximal viscosity threshold was increased from 80 mg/ml (for AB-001) to over 160 mg/ml, effectively doubling the deliverable dose without any impact on activity or changing the formulation. Additional variant R2-020 can extend this further to 185 mg/ml, albeit with a modest 3X loss in binding affinity (from 94.8 pM to 298.7 pM). Of the 39 variants designed, the majority retained binding with slow off-rates, indistinguishable from the parental. The availability of an x-ray co-crystal structure complex allowed us to carefully select mutations that were predicted to be outside the binding paratope. Given the low success rate of mutations that were able to reduce viscosity and maintain the affinity, this was a key component to the method and allowed for a reduction in the total space that needs to be searched. Previous work has shown that key residues/CDR loops can be mutated to have a significant effect on the viscosity. In each of these cases the designs relied on homology models or crystal structure of the FAB only, and were not able to account for residues that were important for binding. [15–18] This is a major limitation in the design/optimization of a therapeutic in that the mutations that can impact viscosity in the CDRs can also have a major impact on activity. Here we are able to strike the balance between the two, leveraging a co-crystal structure of PDGF-BB in complex with a related version of AB-001 in a different framework, and maintain the activity and other biophysical properties of the molecule while achieving these same goals of viscosity reduction, suggesting that the added level of precision in therapeutic design can potentially be achieved through the use of related co-crystal structure in combination with homology modeling.

Several mutations were identified that allowed for the reduction in viscosity, but the majority of mutations had no effect on the overall viscosity of the molecule. Moreover, many of these mutations that had no effect were quite similar to those that had an effect. Each had a net increase in the overall charge, many of them were targeted around negative charge patches and some even made mutations from within the negative charge patches. The fact that some of the mutations altered the viscosity suggest that charge does play an important role in the self-association of viscosity, but the varying effect of the similar mutations suggests that there is more to this problem. Our work here provided the most comprehensive study exploring a wide sampling of charge changes in the Fv and CDR regions along with targeting the multiple negative charge patches. This resulted in the identification of certain “hotspots” of charge patches that drive high viscosity, while similar charge patches located nearby play minimal or opposite role in viscosity. This result came initially as a surprise during the Round 1 study which led to some concerns that LC mutation of N53K somehow was an outlier. However, the additional variants in R2 in the vicinity of LC site N53 provided additional confirmation of the significance of this particular charge patch over other charge patches and regions in Fv. These results indicate that the precise three-dimensional arrangements of the charge patch and the positive charge repulsive mutations play an important role in self-association of the antibodies. It remains to be seen if this type of charge-based viscosity “hotspot” can be generalized with additional viscous antibodies.

Previous work has suggested that charge patterns and asymmetry play an important role in the alteration of viscosity. [18–20] Here we can see that this asymmetry of the charges comes down to the individual residue level. Models that predict only using changes in charge at the domain level are not fine grain enough to differentiate the changes in viscosity that are seen here. This can be exemplified by comparing the SCM score to the Fv charge, Sharma Score and Tomar Score. Here the mutations in the first round had many similarities in terms of charge change in the variable domain or the VH and VL domains and so it was not surprising that only the SCM method could differentiate the successful mutants that target the LC charge patch as opposed to the entire Fv. However, in the R2 designs even this method comes up short as many of these mutations surrounding the remaining LC charge patch have similar effect based upon the SCM score but appear to have very different impacts on the self-association and viscosity of the antibody. Given the differences in antibody charge distributions and the unknown nature of which other part of the antibody this presumed LC charge patch interacts with makes the prediction of the effect of antibody mutants a quite challenging problem. This suggests that this type of prediction of local charge change variants cannot be adequately addressed by methods that are trained only using descriptors that capture only the bulk charge differences between viscous and non-viscous antibodies. This type of training will likely take the type of systematic mutational data described here but done in the context of several different antibody systems. Besides the need for additional data, possible improvements to methods that may be explored are more fine-grain modeling using atomistic rather than domain level or residue level calculations, better descriptions of spatial orientation of charges including dipoles and electrostatic potential surface maps, and predictions of self-association and complementarity within the entire antibody. For therapeutic lead optimization these types of more advanced methods will be needed to be developed.

## Methods

### Structural modeling

The homology model of the Fab region of the human IgG1 antibody AB-001 (VH = EVQLLESGGGLVQPGGSLRLSCAASGFTFSSYAMSWVRQAPGKGLEWVSYISDDGSLKYYADSVKGRFTISRDNSKNTLYLQMNSLRAEDTAVYYCAKHPYWYGGQLDLWGQGTLVTVSS, VL = SYELTQPPSVSVSPGQTASITCSGDSLGSYFVHWYQQKPGQSPVLVIYDDSNRPSGIPERFSGSNSGNTATLTISGTQAMDEADYYCSAFTHNSDVFGGGTKLTVL, CH1 = IGHG1*01 and CL = IGLC2*01) was generated using the Modeler package from Discovery Studio 3.5 and the PDB crystal structure 4QCI as a temple. [27] This structure contained a close homolog of AB-001 (same CDR residues with few Framework mutations) bound to PDGF-BB. For this structure the charge assignment was generated using the “Calculate Protein Ionization and Residue pK” package from Discovery Studio 3.5 (Accelrys Inc.). To prepare the structure for stability and affinity calculations, the dsv formatted structure was constructed by applying the “Prepare Protein” protocol of Discovery Studio 3.5 to the stability and binding affinity input structures with default parameters and the CHARMPLR forcefield.

The change in binding affinity upon mutation was calculated by applying the “Calculate Mutation Energy (Binding)” protocol from Discovery Studio 3.5 to the dsv formatted structure. The default parameters of Forcefield (CHARMm Polar), Internal Dielectric Constant (10), Solvent Dielectric Constant (80), Electrostatic Scaling factor (0.5), VDW Scaling factor (0.5), and Entropy Scaling factor (0.8) were used, with the exception of the Non-polar Surface Coefficient which was set to 0.007. The ligand was defined as the two PDGF-B chains. The change in stability upon mutation was calculated by applying the “Calculate Mutation Energy (Stability)” protocol from Discovery Studio 3.5 to the dsv formatted Fab structure. The default parameters of the Forcefield (CHARMm Polar), Internal Dielectric Constant (10), Solvent Dielectric Constant (80), Electrostatic Scaling factor (0.5), VDW Scaling factor (0.5), and Entropy Scaling factor (0.8) were used, with the exception of the Non-polar Surface Coefficient, which was set to 0.007.

Charge patches were identified by calculating the electrostatic potential surface using the Poison Boltzmann program DelPhi [30–32] which is part of the Discovery Studio 3.5 protocol “Electrostatic Calculations with Focusing”. The parameters for this calculation included 65 grid points per axis, 50% of the coarse grid filled by solute, 90% for the fine grid filled by solute, 0.145 M Salt concentration, solvent dielectric of 80, solute dielectic of 2, solvent probe radius of 1.4 Å and ion exclusion radius of 2.0Å. The electrostatic surface was then visualized using Pymol [36] by loading the DelPhi grid file into an emap and coloring the surface with the range of the electrostatic levels set to -7 (red) to 7 (blue).

### Charge mutations statistics

From the sets of human antibody sequences found in the PDB and IMGT [37–44] all Kabat positions were evaluated for their distribution of amino acid types. Sites were then classified by those which had a high probability of a positive residue (Lys or Arg) (> 10%) in human antibody sequences or sites with a wild-type Glu or Asp that had a high probability of another neutral or charged residue (>10%).

### Computational viscosity prediction

Three methods were utilized to predict the viscosity of antibodies based upon sequence and structural information. The Sharma methods was implemented by following the protocol described in Sharma et al. calculating the viscosity based upon Eq 1, where the viscosity (η) is related to the net charge of the Fv (q), the charge asymmetry (qsym) and the hydrophobic index (HI). [20]

$$\eta =10^{0.15+1.26\times HI-0.043\times q-0.02\times qsym}$$ (1)

Briefly, the net charge of the VH and VL sequences at pH 5.5 were calculated by summing the predicted charges of Asp, Glu, His, Lys and Arg residues, whose charges were defined by the Henderson-Hasselbach equation with pKa values from Berg et al. [45]: Asp (3.9), Glu (4.3), His (6.0), Lys 10.8, and Arg (12.5). The sum of the VH and VL gave the net charge. The charge asymmetry (qsym) was calculated as the product of the VH and VL net charges. Finally, The Hydrophobic index (HI) was calculated as the ratio of sum hydrophobicity of the hydrophobic residues divided by the sum of the hydrophobicity of the hydrophilic residues based upon the Eisenberg scale for each amino acid. [46] Here the hydrophobic residues and scores were: Ala (0.62), Cys (0.29), Phe (1.19), Gly (0.48), Ile (1.38), Leu (1.06), Met (0.64), Pro (0.12), Val (1.08), Trp (0.81), and Tyr (0.26), and the hydrophilic residues and scores were: Asp (-0.90), Glu (-0.74), His (-0.40), Lys (-1.50), Asn (-0.78), Gln (-0.85), Arg (-2.53), Ser (-0.18), and Thr (-0.05).

The Tomar method was implemented by following the protocol described in Tomar et al. for the prediction of the viscosity slope parameter B. [21] Briefly, homology models of the full length IgG were generated using the protein modeler tool in the molecular modeling software package Moe [47] with Fv templates using the homology models of the charge mutants, and full IgG1 template using PDB structure 1HZH. These models were protonated to 5.8 with a salt concentration of 10 mM. The VH-charge, VL-Charge, Hinge-charge and hydrophobic solvent accessible surface area were all calculated with the Moe “Protein Properties” module [47] with the pH set to 5.8 and the salt concentration to 10mM. Using these calculated values we can then use Eq 1 from Tomar et al. [21] to calculate the value B which was used to predict the relative viscosity of the charge mutants.

The SCM method was implemented using the protocol described in Agrawal et al. [19] The SCM protocol (version 3.1.2013) was obtained from the authors and executed with VMD (version 1.9.1) using homology models of the FV regions of the charge mutant antibodies and histidine residues set as neutral HSD residues.

### Protein production

Clarified conditioned transient HEK293 media was loaded onto a protein A affinity column (MabSelect SuRe, GE Healthcare Life Sciences). The protein A column was equilibrated with 50 mM Tris, 150 mM NaCl, pH 7.5. After loading, the column was washed with equilibrium buffer followed by additional wash with 2M Arginine, 50mM Tris pH 7.5 50 mM Tris, 0.5 M CaCl2 pH 7.5. The column was then washed with 10 mM Tris, 10 mM NaCl pH 7.5. The bound protein was then step eluted with 150 mM Glycine, pH 3.5. The protein A pool titrated to pH 7.5 with 2 M Tris base and 0.2 μM filtered. The titrate was then loaded onto a TMAE column (EMD Milipore). The TMAE column was equilibrated and washed with 50mM HEPES, 65mM NaCl pH7.0. Flow-through fractions containing the pure protein of interest were collected. The TMAE pool was mixed with 0.15M NaPO4, 3.75M NaCl, and 3M urea, and then loaded onto a 400ml Buty-S Sepharose 6 Fast Flow (GE Healthcare Life Sciences). The column was washed with 50mM NaPO4, 1.25M NaCl, 1M Urea, pH 7.2 (5-8CV). The flow through was collected and the column was eluted with 50mM NaPO4, 1M Urea, pH 7.2. The HIC pool was buffer exchanged with a Sephadex G-25 Coarse column (GE Healthcare Life Sciences) equilibrated with 10mM Histidine, 5% Sucrose, pH 6.0. The final pool was filtered, concentrated to >50 mg/ml using a 30kDa MWCO device.

### Analytical size exclusion chromatography (aSEC)

Aggregation state of the pre- and post-concentrated viscosity samples were determined by aSEC. Samples were injected onto a YMC-Pack Diol-200 column (300 x 8.0 mm, pore size 200 Å) connected to the Agilent 1260 HPLC system (Agilent Technologies, Santa Clara, CA) with 20 mM sodium phosphate, 400 mM arginine at pH 7.2 as the mobile phase. An isocratic program at a flow rate of 0.75 mL/min for 20 minutes was applied to the aSEC column for sample elution and the data was analyzed using the Agilent OpenLAB Data Analysis software to integrate and quantify peak area of aggregate, protein of interest and low molecular weight species.

### Capillary differential scanning calorimetry (DSC)

Thermal stability of the anti-PDGF-BB viscosity mutants was analyzed using MicroCal’s capillary VP-DSC equipped with an autosampler (Northampton, MA). Protein sample at concentration of 2μM in Histidine/sucrose pH 5.8 buffer was loaded in the sample cell with respective buffer in reference cell. Both the sample and reference cells were heated from 10°C to 110°C at a scan rate of 100°C per hour. The heat capacity difference between the sample cell and reference cell was recorded and analyzed using Origin7.0 software from MicroCal. A baseline thermogram was also generated with Histidine/sucrose pH 5.8 buffer and data was used to subtract any system heat not associated with protein denaturation.

### Viscosity measurements

Protein samples were concentrated to a target of 170 mg/ml using 50 kDa molecular weight cut-off Amicon centrifugal filter units (EMD Millipore, Billerica, MA). For each protein, a set of samples ranging from 25–160 mg/ml were serially diluted using Histidine-sucrose pH 5.8 buffer as diluent. Protein concentrations were determined by 280nm analysis on the SoloVPE Variable Pathlength System (C Technologies, Inc, Bridgewater, NJ). Viscosity measurements were performed using the CP25-1 cone and plate on the MCR-302 rheometer (Anton Paar USA Inc., Ashland, VA) at a constant rotational speed of 150rpm at 25°C. A total of 10 measurements of 10 seconds each were collected per sample and the data was analyzed using the Rheoplus (Anton Paar USA Inc.) V 3.62 software.

### Viscosity parameter fitting

To fit the viscosity data to all ranges of concentration we used the Ross and Minton equation (Eq 2). [26]

$$\eta =\eta_{o}{exp}^{\left(\frac{[\eta ]c}{1-\frac{k}{v}[\eta ]c}\right)}$$ (2)

Here, *η* is the viscosity, *η*_0_ is the viscosity of solution at zero solute concentration, [η] is the intrinsic viscosity, c is the antibody concentration, (k) is the crowding factor and (v) is the shape-determining factor. We used 1.1 cP for *η*_0_ and fit the values of [η] and the ratio (k/v) to the experimental concentration dependent viscosity measurements. This fitting was done using non-linear least-square regression method *nlinfit* in Matlab (R2010b) [48]. From this we were able to interpolate or extrapolate the viscosity at concentrations of 100 mg/ml and 150 mg/ml. We can calculate the 95 percent confidence of this non-linear least-squared fit using the Matlab (R2010b) function *nlpredci* [48].

Eq 2 can also be rearranged to solve for concentration (Eq 3) allowing for us to fit the concentration at which the antibody reaches a specific viscosity.

$$c=\frac{ln(\frac{\eta }{\eta_{o}})}{\left[\eta ](1-\frac{k}{v\;}\text{ln}\left(\frac{\eta }{\eta_{o}}\right)\right)}$$ (3)

Using the same Matlab non-linear least squares fitting functions as before we can fit the concentration where the viscosity reaches 20 cP and the 95% confidence interval for the fit at this value.

### Biacore measurements

All antibodies were off-rate triaged using surface plasmon resonance (SPR) and a Biacore T200 instrument (GE Healthcare, Marlborough, MA). The GE Healthcare anti-human IgG capture antibody was directly immobilized onto a CM5 sensor chip using amine coupling (Amine Coupling Kit, GE Healthcare) according to the manufacturer’s recommendations. The density of the anti-human IgG capture antibody ranged from ~9,000 to 10,000 response units (RUs). The engineered antibodies were diluted to 0.25ug/mL with HBS-EP+ buffer (10mM HEPES, 150mM NaCl, 3mM EDTA, 0.05% Tween20, pH7.4) and captured with anti-human IgG for 44sec at a flow rate of 10uL/min, resulting in capture levels that ranged from ~70 to 100RUs. The human PDGF-BB (PeproTech, Catalog 100–148) was diluted to a single concentration of 2.5nM with HBS-EP+ N buffer (10mM HEPES, 500mM NaCl, 3mM EDTA, 0.05% Tween20, pH7.4) and injected for 170sec at 100uL/min, followed by a dissociation of 300sec. Both the running and sample buffers were HBS-EP+ N and all experiments were performed at 25°C with a data collection rate of 1Hz. The sensor chip surface was regenerated with 3 pulses of 3M MgCl2, 30sec at 50uL/min. All sensorgrams were double referenced with buffer injections against the anti-human IgG control surface. [49] The off-rates were determined with Biacore T200 Evaluation software version 3.0 and BIAevaluation version 4.1.1 (GE Healthcare).

The binding affinities were determined for the parental antibody AB-001 and 4 additional engineered antibodies (R1-016, R2-001, R2-006 and R2-020). Similar to the off-rate screen, SPR and a Biacore T200 instrument was used to measure the off-rate, and in addition the on-rate kinetic constants. Experimental exceptions were the two-fold dilution series of 20, 10, 5, 2.5, and 1.25 nM human PDGF-BB and the analyte injection was shortened to 50sec. The resulting sensorgrams were fit to a 1:1 Langmuir model using Biacore T200 Evaluation software version 3.0 and the K_D_ values were calculated using the equation K_D_ = kd (1/s)/ka (1/Ms).

### Non-specificity assays

DNA ELISA, Insulin Elisa and AC-SINS assays were conducted to determine the change in non-specificity associated with mutations to AB-001. These assays were performed as described in Avery et al. [35] DNA- and insulin-binding scores were calculated as the ratio of the ELISA signal of the antibody at 10 μg/ml to the signal of a well containing buffer instead of the primary antibody. For AC-SINS the smoothed max absorbance of the average blank (PBS) was subtracted from the smoothed max absorbance of the antibody sample to determine the antibody AC-SINS score. For each the DNA, Insulin and AC-SINS assays, scores ≥11 are considered high based upon their correlation with in vivo PK data. [35]

## Supporting information

S1 Fig Viscosity analysis of anti-PDGF-BB antibody AB-001. The viscosities of AB-001 in 20 mM histidine buffer was measured using a DLS bead-based method with no-salt (blue), 150nM NaCl (red) and 50 mM Arg (green) and plotted against the concentration of the samples. These are compared to the desired formulation viscosity of 20 cP shown in the dashed black line. See Supplemental Methods for a description of viscosity measurements.
(DOCX)

S2 Fig Fitting of viscosity (cP) versus concentration (mg/ml) and concentration (mg/ml) versus ln(viscosity(cP)) using the Ross Minton equation. Curve fit include variable parameters [η] and k/v. The fit to the curve is determined by calculating the correlation coefficient (R^2^).
(DOCX)

S1 Table List of heavy chain and light chain variable regions for designed sequences. Sequences are compared to the parental AB-001 sequence.
(DOCX)

S2 Table List of mutants with sites indicated in Kabat notation, predicted stability, predicted affinity and AA frequency. (DOCX)

S3 Table Viscosity measurements for designed antibodies. For a series of different concentrations for each designed antibody, the concentration with standard deviation (n = 2 measurements except for * where n = 1) are shown in mg/ml and the viscosity measurements are shown in cP.
(DOCX)

## Abbreviations

aSEC: analytical size exclusion chromatography
CDR: complementarity-determining region
cP: centipoise
DSC: differential scanning calorimetry
Fv: fragment variable region
FW: framework region
HC: heavy chain
HMMS: high molecular mass species
K_D_: dissociation constant
LC: light chain
LMMS: low molecular mass species
PDB: Protein Databank
PDGF-B: platelet derived growth factor B
PDGF-BB: platelet derived growth factor B homodimer
RMSD: root mean squared deviation
SC: subcutaneous
SCM: Spatial Charge Map
SDR: specificity-determining residues
SPR: surface plasmon resonance
VH: variable heavy chain
VL: variable light chain
