# Supplementary material for: Modeling and mitigation of high-concentration antibody viscosity through structure-based computer-aided protein design
Source: PLoS One. 2020 May 7;15(5):e0232713. doi: 10.1371/journal.pone.0232713 (PMC7205207; doi:10.1371/journal.pone.0232713)
Supplement: S1 Fig — The viscosities of AB-001 in 20 mM histidine buffer was measured using a DLS bead-based method with no-salt (blue), 150nM NaCl (red) and 50 mM Arg (green) and plotted against the concentration of the samples. These are compared to the desired formulation viscosity of 20 cP shown in the dashed black line. See Supplemental Methods for a description of viscosity measurements. (DOCX) [file pone.0232713.s001.docx]

**Fig S1. Viscosity analysis of anti-PDGF Antibody AB-001**. The viscosities of AB-001 in 20 mM histidine buffer was measured using a DLS bead-based method with no-salt (blue), 150nM NaCl (red) and 50 mM Arg (green) and plotted against the concentration of the samples. These are compared to the desired formulation viscosity of 20 cP shown in the dashed black line. See Supplemental Methods for a description of viscosity measurements.

**S1 Fig Supporting Method**

Viscosity Measurements of AB-001 with added Salt

Transient expression of antibodies was performed in HEK-293F cells. Cell culture supernatant was harvested three to seven days after transfection and cleared by centrifugation. After filtration (0.22 μm or 0.45 μm), the supernatant was subjected to standard protein A or G affinity chromatography (MabSelect SURE, GE). Proteins were eluted at pH 3 and neutralized in 3 M TRIS, pH 8. Further downstream processing involved buffer exchange to 1× Dulbecco's PBS (Invitrogen) and sterile filtration (0.2 μm; Millipore or Sartorius). Purity was analyzed under denaturing, reducing and denaturing, non-reducing conditions in SDS-PAGE or by capillary electrophoresis. HP-SEC was performed to analyze IgG preparations in their native state.

Viscosity of AB-001 was measured as follows. Proteins in PBS were extensively dialyzed against 20 mM histidine, 85 mg/ml sucrose, 0.05 mg/ml EDTA pH 6.0 and no salt, 150 NaCl or 50 mM L-Arginine using membrane cassette devices 10K MWCO (Thermo Scientific). Proteins were harvested from dialysis and filtered using a 0.2-micron syringe filter. Proteins were concentrated using Vivaspin centrifugal concentrators 10K MWCO (GE Healthcare). Sample aliquots (12 μl) were removed from the concentrator retentate as the protein volume was reduced and the protein concentration increased. 300 nm beads (Nanosphere, Thermo Scientific) were added to the protein samples and buffer blank. The beads were diluted 1:10 in 20 mM histidine, 85 mg/ml sucrose, 0.05 mg/ml EDTA pH 6.0 and 0.75 μl diluted beads were spiked into the protein sample. The protein/bead and buffer/bead samples were mixed by gently vortexing. 8 μl sample was transferred to 1536 well plate (SensoPlate, glass bottom, Greiner Bio-One) for analysis by dynamic light scattering measurements (DLS). The plate was sealed with optically clear tape and centrifuged at 2000 RPM for 2 minutes to remove bubbles.

The DLS measurements were made using a DynaPro Plate Reader (Wyatt Technology, Santa Barbara, Calif.). Samples were incubated at 25° C. and measured with 15 consecutive 25 second acquisitions. Radius of the bead was averaged for data acquisitions that had acceptable decay curves. The viscosity was calculated based on the Stokes-Einstein equation. Sample viscosity was calculated as the measured apparent radius divided by the nominal bead radius times 0.893 cP, the viscosity of water at 25° C.

1. Ross PD, Minton AP. Hard quasispherical model for the viscosity of hemoglobin solutions. Biochem Biophys Res Commun 1977; 76:971-6.
