# Supplementary material for: Modeling and mitigation of high-concentration antibody viscosity through structure-based computer-aided protein design
Source: PLoS One. 2020 May 7;15(5):e0232713. doi: 10.1371/journal.pone.0232713 (PMC7205207; doi:10.1371/journal.pone.0232713)
Supplement: S2 Fig — Curve fit include variable parameters [η] and k/v. The fit to the curve is determined by calculating the correlation coefficient (R2). (DOCX) [file pone.0232713.s002.docx]

**S2 Fig: Fitting of viscosity (cP) versus concentration (mg/ml) and concentration (mg/ml) versus ln(viscosity(cP)) using the Ross Minton Equation**. Curve fit include variable parameters [η] and k/v. The fit to the curve is determined by calculating the correlation coefficient (R^2^).

AB-001

|  |  |
| --- | --- |

R1-002

|  |  |
| --- | --- |

R1-003

|  |  |
| --- | --- |

R1-004

|  |  |
| --- | --- |

R1-005

|  |  |
| --- | --- |

R1-006

|  |  |
| --- | --- |

R1-007

|  |  |
| --- | --- |

R1-008

|  |  |
| --- | --- |

R1-009

|  |  |
| --- | --- |

R1-010

|  |  |
| --- | --- |

R1-011

|  |  |
| --- | --- |

R1-012

|  |  |
| --- | --- |

R1-013

|  |  |
| --- | --- |

R1-014

|  |  |
| --- | --- |

R1-015

|  |  |
| --- | --- |

R1-016

|  |  |
| --- | --- |

R1-017

|  |  |
| --- | --- |

R1-018

|  |  |
| --- | --- |

R2-001

|  |  |
| --- | --- |

R2-004

|  |  |
| --- | --- |

R2-005

|  |  |
| --- | --- |

R2-006

|  |  |
| --- | --- |

R2-007

|  |  |
| --- | --- |

R2-008

|  |  |
| --- | --- |

R2-009

|  |  |
| --- | --- |

R2-010

|  |  |
| --- | --- |

R2-011

|  |  |
| --- | --- |

R2-012

|  |  |
| --- | --- |

R2-013

|  |  |
| --- | --- |

R2-014

|  |  |
| --- | --- |

R2-015

|  |  |
| --- | --- |

R2-016

|  |  |
| --- | --- |

R2-017

|  |  |
| --- | --- |

R2-018

|  |  |
| --- | --- |

R2-019

|  |  |
| --- | --- |

R2-020

|  |  |
| --- | --- |

R2-021

|  |  |
| --- | --- |

R2-022

|  |  |
| --- | --- |
