# Supplementary material for: Modeling and mitigation of high-concentration antibody viscosity through structure-based computer-aided protein design
Source: PLoS One. 2020 May 7;15(5):e0232713. doi: 10.1371/journal.pone.0232713 (PMC7205207; doi:10.1371/journal.pone.0232713)
Supplement: S1 Table — Sequences are compared to the parental AB-001 sequence. (DOCX) [file pone.0232713.s003.docx]

**Table S1a: List of heavy chain variable regions for designed sequences.** Sequences are compared to the parental AB-001 sequence.

| **Name** | **FW1** | **CDR1** | **FW2** | **CDR2** | **FW3** | **CDR3** | **FW4** |
| --- | --- | --- | --- | --- | --- | --- | --- |
| AB-001 | EVQLLESGGGLVQPGGSLRLSCAAS | GFTFSSYAMS | WVRQAPGKGLEWVS | YISDDGSLKYYADSVKG | RFTISRDNSKNTLYLQMNSLRAEDTAVYYCAK | HPYWYGGQLDL | WGQGTLVTVSS |
| 4QCI | ----V-------------------- | ---------- | -------------- | ----------------- | -------------------------------R | ----------- | ----------- |
| R1-002 | -----Q------------------- | ---------- | -------------- | ----------------- | -------------------------------R | ----------- | ----------- |
| R1-003 | ------------K------------ | ---------- | -------------- | ----------------- | -------------------------------R | ----------- | ----------- |
| R1-004 | ----------------------R-- | ---------- | -------------- | ----------------- | -------------------------------R | ----------- | ----------- |
| R1-005 | ------------------------- | ---------- | -------------- | ---N------------- | -------------------------------R | ----------- | ----------- |
| R1-006 | ------------------------- | ---------- | -------------- | ----------------- | -------------------------------R | ----------- | --R-------- |
| R1-007 | -----Q------K------------ | ---------- | -------------- | ----------------- | -------------------------------R | ----------- | ----------- |
| R1-008 | ------------------------- | ---------- | -------------- | ----------------- | -------------------------------- | ----------- | ----------- |
| R1-009 | ------------------------- | ---------- | -------------- | ----------------- | -------------------------------- | ----------- | ----------- |
| R1-010 | ------------------------- | ---------- | -------------- | ----------------- | -------------------------------- | ----------- | ----------- |
| R1-011 | ------------------------- | ---------- | -------------- | ----------------- | -------------------------------- | ----------- | ----------- |
| R1-012 | -----Q------K------------ | ---------- | -------------- | ----------------- | -------------------------------R | ----------- | ----------- |
| R1-013 | -----Q------------------- | ---------- | -------------- | ----------------- | -------------------------------R | ----------- | ----------- |
| R1-014 | -----Q------K------------ | ---------- | -------------- | ----------------- | -------------------------------R | ----------- | ----------- |
| R1-015 | -----Q------K------------ | ---------- | -------------- | ----------------- | -------------------------------R | ----------- | ----------- |
| R1-016 | -----Q------K------------ | ---------- | -------------- | ----------------- | -------------------------------R | ----------- | ----------- |
| R1-017 | ------------K------------ | ---------- | -------------- | ----------------- | -------------------------------R | ----------- | ----------- |
| R1-018 | ------------------------- | ---------- | -------------- | ----------------- | -------------------------------R | ----------- | --R-------- |
| R2-001 | -----Q------K------------ | ---------- | -------------- | ----------------- | -------------------------------R | ----------- | ----------- |
| R2-002 | -----Q------K------------ | ---------- | -------------- | ----------------- | -------------------------------R | ----------- | ----------- |
| R2-003 | -----Q------K------------ | ---------- | -------------- | ----------------- | -------------------------------R | ----------- | ----------- |
| R2-004 | -----Q------K------------ | ---------- | -------------- | ----------------- | -------------------------------R | ----------- | ----------- |
| R2-005 | -----Q------K------------ | ---------- | -------------- | ----------------- | -------------------------------R | ----------- | ----------- |
| R2-006 | -----Q------K------------ | ---------- | -------------- | ----------------- | -------------------------------R | ----------- | ----------- |
| R2-007 | -----Q------K------------ | ---------- | -------------- | ----------------- | -------------------------------R | ----------- | ----------- |
| R2-008 | -----Q------K------------ | ---------- | -------------- | ----------------- | -------------------------------R | ----------- | ----------- |
| R2-009 | -----Q------K------------ | ---------- | -------------- | ----------------- | -------------------------------R | ----------- | ----------- |
| R2-010 | -----Q------K------------ | ---------- | -------------- | ----------------- | -------------------------------R | --H-------- | ----------- |
| R2-011 | -----Q------K------------ | ---------- | -------------- | ----------------- | -------------------------------R | -------K--- | ----------- |
| R2-012 | -----Q------K------------ | ---------- | -------------- | ---K------------- | -------------------------------R | ----------- | ----------- |
| R2-013 | -----Q------K------------ | ---------- | -------------- | ---N------------- | -------------------------------R | ----------- | ----------- |
| R2-014 | -----Q------K------------ | ---------- | -------------- | ----Q------------ | -------------------------------R | ----------- | ----------- |
| R2-015 | -----Q------K------------ | ---------- | -------------- | ------------N---- | -------------------------------R | ----------- | ----------- |
| R2-016 | -----Q------K------------ | ---------- | -------------- | ----------------- | -------------------------------R | ---------N- | ----------- |
| R2-017 | -----Q------K------------ | ---------- | -------------- | ----------------- | -------------------------------R | ---------Y- | ----------- |
| R2-018 | -----Q------K------------ | ---------- | -------------- | ----------------- | -------------------------------R | ----------- | ----------- |
| R2-019 | -----Q------K------------ | ---------- | -------------- | ----------------- | -------------------------------R | ----------- | ----------- |
| R2-020 | -----Q------K------------ | ---------- | -------------- | ----------------- | -------------------------------R | ----------- | ----------- |
| R2-021 | -----Q------K------------ | ---------- | -------------- | ----------------- | -------------------------------R | ----------- | ----------- |
| R2-022 | -----Q------K------------ | ---------- | -------------- | ----------------- | -------------------------------R | ----------- | ----------- |

**Table S1b: List of light chain variable regions for designed sequences**. Sequences are compared to the parental AB-001 sequence.

| **Name** | **FW1** | **CDR1** | **FW2** | **CDR2** | **FW3** | **CDR3** | **FW4** |
| --- | --- | --- | --- | --- | --- | --- | --- |
| AB-001 | SYELTQPPSVSVSPGQTASITC | SGDSLGSYFVH | WYQQKPGQSPVLVIY | DDSNRPS | GIPERFSGSNSGNTATLTISGTQAMDEADYYC | SAFTHNSDV | FGGGTKLTVL |
| 4QCI | ------------A-----R-S- | ----------- | --------A------ | ------- | ------------------------E------- | --------- | ---------- |
| R1-002 | ---------------------- | ----------- | --------------- | ------- | -------------------------------- | --------- | ---------- |
| R1-003 | ---------------------- | ----------- | --------------- | ------- | -------------------------------- | --------- | ---------- |
| R1-004 | ---------------------- | ----------- | --------------- | ------- | -------------------------------- | --------- | ---------- |
| R1-005 | ---------------------- | ----------- | --------------- | ------- | -------------------------------- | --------- | ---------- |
| R1-006 | ---------------------- | ----------- | --------------- | ------- | -------------------------------- | --------- | ---------- |
| R1-007 | ---------------------- | ----------- | --------------- | ------- | -------------------------------- | --------- | ---------- |
| R1-008 | --V------------------- | ----------- | --------------- | ------- | -------------------------------- | --------- | ---------- |
| R1-009 | ----------------R----- | ----------- | --------------- | ------- | -------------------------------- | --------- | ---------- |
| R1-010 | ---------------------- | ----------- | --------------- | ---K--- | -------------------------------- | --------- | ---------- |
| R1-011 | ---------------------- | ----------- | --------------- | ------- | -------------------------------- | -------N- | ---------- |
| R1-012 | --V------------------- | ----------- | --------------- | ------- | -------------------------------- | --------- | ---------- |
| R1-013 | ----------------R----- | ----------- | --------------- | ------- | -------------------------------- | --------- | ---------- |
| R1-014 | ----------------R----- | ----------- | --------------- | ------- | -------------------------------- | --------- | ---------- |
| R1-015 | ---------------------- | ----------- | --------------- | ------- | -------------------------------- | -------N- | ---------- |
| R1-016 | ---------------------- | ----------- | --------------- | ---K--- | -------------------------------- | --------- | ---------- |
| R1-017 | ----------------R----- | ----------- | --------------- | ------- | -------------------------------- | --------- | ---------- |
| R1-018 | ----------------R----- | ----------- | --------------- | ------- | -------------------------------- | --------- | ---------- |
| R2-001 | --V---------A--K--R--- | ----------- | --------------- | ---K--- | -------------------------------- | --------- | ---------- |
| R2-002 | --V---------A--K--R--- | ---K------- | --------------- | ---K--- | -------------------------------- | --------- | ---------- |
| R2-003 | --V---------A--K--R--- | ----------K | --------------- | ---K--- | -------------------------------- | --------- | ---------- |
| R2-004 | --V---------A--K--R--- | ----------- | --------------H | ---K--- | -------------------------------- | --------- | ---------- |
| R2-005 | --V---------A--K--R--- | ----------- | --------------R | ---K--- | -------------------------------- | --------- | ---------- |
| R2-006 | --V---------A--K--R--- | ----------- | --------------- | --KK--- | -------------------------------- | --------- | ---------- |
| R2-007 | --V---------A--K--R--- | ----------- | --------------- | ---K--- | --------K----------------------- | --------- | ---------- |
| R2-008 | --V---------A--K--R--- | ----------- | --------------- | ---K--- | ----------K--------------------- | --------- | ---------- |
| R2-009 | --V---------A--K--R--- | ----------- | --------------- | ---K--- | -----------K-------------------- | --------- | ---------- |
| R2-010 | --V---------A--K--R--- | ----------- | --------------- | ---K--- | -------------------------------- | --------- | ---------- |
| R2-011 | --V---------A--K--R--- | ----------- | --------------- | ---K--- | -------------------------------- | --------- | ---------- |
| R2-012 | --V---------A--K--R--- | ----------- | --------------- | ---K--- | -------------------------------- | --------- | ---------- |
| R2-013 | --V---------A--K--R--- | ----------- | --------------- | ---K--- | -------------------------------- | --------- | ---------- |
| R2-014 | --V---------A--K--R--- | ----------- | --------------- | ---K--- | -------------------------------- | --------- | ---------- |
| R2-015 | --V---------A--K--R--- | ----------- | --------------- | ---K--- | -------------------------------- | --------- | ---------- |
| R2-016 | --V---------A--K--R--- | ----------- | --------------- | ---K--- | -------------------------------- | --------- | ---------- |
| R2-017 | --V---------A--K--R--- | ----------- | --------------- | ---K--- | -------------------------------- | --------- | ---------- |
| R2-018 | --V---------A--K--R--- | --N-------- | --------------- | ---K--- | -------------------------------- | --------- | ---------- |
| R2-019 | --V---------A--K--R--- | ----------- | --------------- | L--K--- | -------------------------------- | --------- | ---------- |
| R2-020 | --V---------A--K--R--- | ----------- | --------------- | -N-K--- | -------------------------------- | --------- | ---------- |
| R2-021 | --V---------A--K--R--- | ----------- | --------------- | ---K--- | -------------------------------- | -------K- | ---------- |
| R2-022 | --V---------A--K--R--- | ----------- | --------------- | ---K--- | -------------------------------- | -------N- | ---------- |
