# Supplementary material for: Modeling and mitigation of high-concentration antibody viscosity through structure-based computer-aided protein design
Source: PLoS One. 2020 May 7;15(5):e0232713. doi: 10.1371/journal.pone.0232713 (PMC7205207; doi:10.1371/journal.pone.0232713)
Supplement: S2 Table — (DOCX) [file pone.0232713.s004.docx]

**S2 Table: List of mutants with sites indicated in Kabat notation, predicted stability, predicted affinity and AA Frequency.**

| Site | WT | Mut | Location | ddG Stability | ddG Affinity | AA Frequency |
| --- | --- | --- | --- | --- | --- | --- |
| H6 | E | Q | HFW1 | -0.30 | 0.08 | 51.71% |
| H13 | Q | K | HFW1 | 0.20 | 0.51 | 63.59% |
| H23 | A | K | HFW1 | -0.98 | 0.61 | 46.44% |
| H23 | A | R | HFW1 | -0.67 | 0.60 | 0.52% |
| H52a | D | N | HCDR2 | -1.21 | 0.43 | 1.70% |
| H100b | Q | R | HCDR3 | -1.44 | 0.89 | 3.78% |
| L3 | E | V | LFW1 | -0.60 | 0.89 | 75.18% |
| L18 | T | R | LFW1 | -0.30 | 0.74 | 35.44% |
| L53 | N | K | LCDR2 | -0.41 | -0.05 | 18.10% |
| L96 | D | N | LCDR3 | -0.56 | 1.10 | 2.18% |
| L49 | Y | H | LFW2 | 0.03 | 0.03 | 1.30% |
| L49 | Y | R | LFW2 | 0.46 | 0.99 | 1.50% |
| L52 | S | K | LCDR2 | -0.16 | -0.53 | 0.41% |
| L65 | S | K | LFW3 | 0.13 | 0.86 | 1.00% |
| L67 | S | K | LFW3 | 0.96 | 0.93 | 0.00% |
| L68 | G | K | LFW3 | 1.08 | 0.91 | 0.00% |
| H97 | Y | H | HCDR3 | 0.08 | 0.39 | 1.13% |
| H100b | Q | K | HCDR3 | -0.29 | 1.16 | 0.29% |
| H52a | D | K | HCDR2 | -1.65 | -1.42 | 0.34% |
| H52a | D | N | HCDR2 | -1.21 | 0.43 | 1.70% |
| H53 | D | Q | HCDR2 | -0.76 | 0.42 | 0.05% |
| H61 | D | N | HCDR2 | -0.80 | 1.30 | 0.22% |
| H101 | D | N | HCDR3 | 0.30 | 0.90 | 2.22% |
| H101 | D | Y | HCDR3 | 0.42 | 0.82 | 0.60% |
| L26 | D | N | LCDR1 | -0.28 | 1.15 | 4.55% |
| L50 | D | L | LCDR2 | -1.26 | 0.66 | 1.13% |
| L51 | D | N | LCDR3 | -1.07 | 1.99 | 26.51% |
| L96 | D | N | LCDR3 | -0.56 | 1.10 | 2.18% |
| L96 | D | K | LCDR3 | -1.35 | 2.06 | 0.51% |
