# Supplementary material for: Modeling and mitigation of high-concentration antibody viscosity through structure-based computer-aided protein design
Source: PLoS One. 2020 May 7;15(5):e0232713. doi: 10.1371/journal.pone.0232713 (PMC7205207; doi:10.1371/journal.pone.0232713)
Supplement: S3 Table — For a series of different concentrations for each designed antibody, the concentration with standard deviation (n = 2 measurements except for * where n = 1) are shown in mg/ml and the viscosity measurements are shown in cP. (DOCX) [file pone.0232713.s005.docx]

**S3 Table. Viscosity measurements for designed antibodies.** For a series of different concentrations for each designed antibody, the concentration with standard deviation (n=2 measurements except for * where n=1) are shown in mg/ml and the viscosity measurements are shown in cP.

| AB-001 | | | |
| --- | --- | --- | --- |
| Conc. (mg/ml) | | | Viscosity (cP) |
| 25.0 | ± | 0.2 | 2.9 |
| 47.9 | ± | 0.7 | 5.2 |
| 57.8 | ± | 1.4 | 7.2 |
| 66.4 | ± | 1.7 | 10.6 |
| 76.8 | ± | 2.1 | 16.0 |
| 90.6 | ± | 2.6 | 26.6 |
| 98.8 | ± | 2.8 | 38.6 |
|  |  |  |  |
| R1-002 | | | |
| Conc. (mg/ml) | | | Viscosity (cP) |
| 24.7 | ± | 0.4 | 2.7 |
| 48.3 | ± | 0.3 | 4.7 |
| 63.7 | ± | 0.4 | 7.2 |
| 73 | ± | 1.6 | 10.3 |
| 82.6 | ± | 2.2 | 16.0 |
| 95.4 | ± | 1.2 | 23.5 |
| 107.6 | ± | 2.6 | 40.2 |
|  |  |  |  |
| R1-003 | | | |
| Conc. (mg/ml) | | | Viscosity (cP) |
| 24.8 | ± | 0.3 | 2.9 |
| 49.3 | ± | 0.1 | 5.1 |
| 58.2 | ± | 1.4 | 7.1 |
| 66.9 | ± | 0.7 | 10.1 |
| 79.6 | ± | 1.9 | 16.4 |
| 88.9 | ± | 1.8 | 24.3 |
|  |  |  |  |
| R1-004 | | | |
| Conc. (mg/ml) | | | Viscosity (cP) |
| 24.5 | ± | 0.4 | 2.9 |
| 49.7 | ± | 0.9 | 5.2 |
| 62 | ± | 0.6 | 7.9 |
| 71.6 | ± | 1.9 | 11.5 |
| 81.6 | ± | 2.1 | 17.5 |
| 91.8 | ± | 2.4 | 27.2 |
| 104.3 | ± | 3.5 | 42.4 |
|  |  |  |  |
| R1-005 | | | |
| Conc. (mg/ml) | | | Viscosity (cP) |
| 25.8 | ± | 0.2 | 1.9 |
| 54.1 | ± | 1.5 | 4.2 |
| 70.6 | ± | 0.9 | 8.0 |
| 82.9 | ± | 0.6 | 12.8 |
| 97 | ± | 0.7 | 24.0 |
| 113.9 | ± | 2.2 | 44.7 |
| 130.7 | ± | 4.0 | 84.5 |
|  |  |  |  |
| R1-006 | | | |
| Conc. (mg/ml) | | | Viscosity (cP) |
| 25.4 | ± | 0.2 | 3.0 |
| 51.3 | ± | 0.8 | 5.8 |
| 69.9 | ± | 1.5 | 12.9 |
| 81.5 | ± | 1.9 | 19.1 |
| 89.7 | ± | 2.1 | 27.8 |
| 100.5 | ± | 2.4 | 43.0 |
| 111.8 | ± | 2.1 | 63.8 |
| 124.3 | ± | 2.8 | 107.8 |
|  |  |  |  |
| R1-007 | | | |
| Conc. (mg/ml) | | | Viscosity (cP) |
| 23.3 | ± | 0.2 | 2.7 |
| 49.5 | ± | 0.8 | 4.4 |
| 71.7 | ± | 1.9 | 8.4 |
| 83.8 | ± | 0.5 | 16.7 |
| 93 | ± | 0.8 | 25.4 |
| 119.7 | ± | 1.6 | 64.4 |
| 128.5 | ± | 3.2 | 98.4 |
| 138.9 | ± | 0.3 | 153.4 |
| 146.8 | ± | 1.9 | 224.9 |
| 156.9 | ± | 5.1 | 295.4 |
|  |  |  |  |
| R1-008 | | | |
| Conc. (mg/ml) | | | Viscosity (cP) |
| 24.6 | ± | 0.0 | 2.4 |
| 48.0 | ± | 0.4 | 3.7 |
| 63.4 | ± | 0.4 | 6.6 |
| 74.6 | ± | 0.7 | 9.8 |
| 86.6 | ± | 1.1 | 16.7 |
| 103.2 | ± | 1.9 | 36.7 |
| 111.1 | ± | 0.2 | 52.3 |
|  |  |  |  |
| R1-009 | | | |
| Conc. (mg/ml) | | | Viscosity (cP) |
| 25.0 | ± | 0.1 | 3.0 |
| 50.4 | ± | 0.5 | 4.7 |
| 57.4 | ± | 0.2 | 6.1 |
| 68.5 | ± | 0.5 | 8.5 |
| 79.6 | ± | 0.5 | 12.3 |
| 89.9* |  |  | 19.4 |
| 102.2 | ± | 0.4 | 32.9 |
|  |  |  |  |
| R1-010 | | | |
| Conc. (mg/ml) | | | Viscosity (cP) |
| 25.5 | ± | 0.1 | 2.7 |
| 49.9 | ± | 0.2 | 3.9 |
| 69.2 | ± | 0.2 | 6.2 |
| 85.4 | ± | 0.3 | 9.6 |
| 100.7 | ± | 0.5 | 16.4 |
| 111.1 | ± | 0.9 | 23.1 |
| 121.0 | ± | 0.4 | 32.5 |
|  |  |  |  |
| R1-011 | | | |
| Conc. (mg/ml) | | | Viscosity (cP) |
| 25.3 | ± | 0.1 | 2.9 |
| 50.0 | ± | 0.2 | 5.9 |
| 66.1 | ± | 0.0 | 12.3 |
| 76.4 | ± | 1.0 | 19.2 |
| 87.5 | ± | 0.5 | 33.7 |
| 98.3 | ± | 0.5 | 55.8 |
| 110.4 | ± | 0.1 | 87.6 |
|  |  |  |  |
| R1-012 | | | |
| Conc. (mg/ml) | | | Viscosity (cP) |
| 25.3 | ± | 0.4 | 3.3 |
| 49.9 | ± | 1.0 | 5.9 |
| 57.7 | ± | 1.2 | 8.1 |
| 69.2 | ± | 0.7 | 11.4 |
| 79.5 | ± | 2.0 | 17.2 |
| 89.1 | ± | 2.5 | 23.1 |
| 109 | ± | 2.4 | 58.9 |
|  |  |  |  |
| R1-013 | | | |
| Conc. (mg/ml) | | | Viscosity (cP) |
| 24.7 | ± | 0.0 | 3.5 |
| 52.1 | ± | 0.4 | 5.4 |
| 57.2 | ± | 0.4 | 6.4 |
| 68.7 | ± | 0.3 | 8.7 |
| 79.0 | ± | 0.5 | 12.1 |
| 89.0 | ± | 0.5 | 18.1 |
| 100.8 | ± | 1.1 | 31.0 |
|  |  |  |  |
| R1-014 | | | |
| Conc. (mg/ml) | | | Viscosity (cP) |
| 24.8 | ± | 0.0 | 3.4 |
| 50.5 | ± | 0.3 | 5.1 |
| 60.2 | ± | 0.7 | 6.9 |
| 69.2 | ± | 4.2 | 8.5 |
| 75.7 | ± | 0.2 | 11.6 |
| 86.5 | ± | 1.1 | 17.8 |
| 94.6 | ± | 0.1 | 22.9 |
| 112.1 | ± | 2.1 | 44.6 |
| 123.5 | ± | 0.6 | 86.9 |
|  |  |  |  |
| R1-015 | | | |
| Conc. (mg/ml) | | | Viscosity (cP) |
| 25.1 | ± | 0.4 | 2.8 |
| 49.6 | ± | 0.8 | 5.3 |
| 57.9 | ± | 0.1 | 7.7 |
| 68 | ± | 1.6 | 11.8 |
| 83.1 | ± | 2.0 | 18.5 |
| 88.5 | ± | 2.2 | 26.9 |
| 104.1 | ± | 2.9 | 49.2 |
|  |  |  |  |
| R1-016 | | | |
| Conc. (mg/ml) | | | Viscosity (cP) |
| 25.7 | ± | 0.2 | 2.8 |
| 50.7 | ± | 0.5 | 4.0 |
| 71.5 | ± | 0.9 | 6.4 |
| 82.8 | ± | 0.3 | 8.8 |
| 99.5 | ± | 1.5 | 14.0 |
| 120.3 | ± | 0.2 | 26.9 |
| 133.3 | ± | 1.5 | 41.7 |
| 156.6 | ± | 1.9 | 92.3 |
|  |  |  |  |
| R1-017 | | | |
| Conc. (mg/ml) | | | Viscosity (cP) |
| 25.7 | ± | 0.0 | 2.7 |
| 50.4 | ± | 0.0 | 4.9 |
| 61.3 | ± | 0.2 | 7.2 |
| 69 | ± | 0.1 | 10.0 |
| 81 | ± | 0.1 | 16.4 |
| 89.4 | ± | 0.8 | 24.7 |
|  |  |  |  |
| R1-018 | | | |
| Conc. (mg/ml) | | | Viscosity (cP) |
| 25 | ± | 0.3 | 2.8 |
| 50 | ± | 0.8 | 5.1 |
| 59.6 | ± | 1.2 | 6.5 |
| 68.3 | ± | 0.5 | 9.2 |
| 79.4 | ± | 1.7 | 13.8 |
| 88 | ± | 0.6 | 20.8 |
| 100.3 | ± | 2.3 | 33.4 |
| 109.7 | ± | 2.5 | 51.3 |
|  |  |  |  |
| R2-001 | | | |
| Conc. (mg/ml) | | | Viscosity (cP) |
| 18.1 | ± | 1.0 | 1.4 |
| 50.7 | ± | 0.4 | 3.1 |
| 82.1 | ± | 1.3 | 5.2 |
| 101.1 | ± | 22.5 | 9.3 |
| 131.8 | ± | 1.1 | 13.6 |
| 149.8 | ± | 0.3 | 39.0 |
| 153.2 | ± | 3.5 | 43.1 |
|  |  |  |  |
|  |  |  |  |
| R2-004 | | | |
| Conc. (mg/ml) | | | Viscosity (cP) |
| 27.8 | ± | 8.1 | 1.9 |
| 45.3 | ± | 5.4 | 3.6 |
| 74.0 | ± | 1.0 | 9.1 |
| 114.2 | ± | 5.6 | 19.9 |
| 125.6 | ± | 0.3 | 29.5 |
| 164.4 | ± | 5.0 | 79.2 |
|  |  |  |  |
|  |  |  |  |
| R2-005 | | | |
| Conc. (mg/ml) | | | Viscosity (cP) |
| 15.2 | ± | 0.3 | 2.6 |
| 36.7 | ± | 6.2 | 4.0 |
| 64.0 | ± | 4.5 | 7.9 |
| 79.9 | ± | 5.1 | 14.5 |
| 140.1 | ± | 0.8 | 24.1 |
| 168.8 | ± | 0.9 | 64.8 |
|  |  |  |  |
| R2-006 | | | |
| Conc. (mg/ml) | | | Viscosity (cP) |
| 13.4 | ± | 0.4 | 2.2 |
| 35.9 | ± | 0.2 | 2.7 |
| 59.5 | ± | 0.4 | 3.8 |
| 74.4 | ± | 0.0 | 4.8 |
| 138.8 | ± | 0.6 | 8.9 |
| 164.2 | ± | 4.8 | 18.9 |
| 173.5 | ± | 10.5 | 27.5 |
|  |  |  |  |
| R2-007 | | | |
| Conc. (mg/ml) | | | Viscosity (cP) |
| 15.1 | ± | 0.2 | 2.9 |
| 38.8 | ± | 3.5 | 3.2 |
| 68.2 | ± | 1.2 | 4.3 |
| 80.1 | ± | 2.3 | 8.4 |
| 116.9 | ± | 18.1 | 13.8 |
| 156.1 | ± | 0.4 | 20.5 |
| 177.2 | ± | 19.6 | 47.4 |
|  |  |  |  |
| R2-008 | | | |
| Conc. (mg/ml) | | | Viscosity (cP) |
| 17.7 | ± | 2.9 | 1.5 |
| 33.9 | ± | 1.2 | 2.0 |
| 67.6 | ± | 4.7 | 3.7 |
| 127.7 | ± | 1.1 | 6.5 |
| 154.2 | ± | 1.2 | 30.2 |
| 179.7 | ± | 9.4 | 57.8 |
|  |  |  |  |
| R2-009 | | | |
| Conc. (mg/ml) | | | Viscosity (cP) |
| 22.6 | ± | 0.1 | 1.7 |
| 41.6 | ± | 0.1 | 2.0 |
| 79.8 | ± | 2.9 | 4.9 |
| 125.4 | ± | 2.5 | 9.6 |
| 154.3 | ± | 0.2 | 23.6 |
| 168.1 | ± | 15.7 | 69.3 |
|  |  |  |  |
|  |  |  |  |
| R2-010 | | | |
| Conc. (mg/ml) | | | Viscosity (cP) |
| 18.2 | ± | 0.4 | 2.6 |
| 74.5 | ± | 0.3 | 6.5 |
| 110.8 | ± | 5.0 | 12.9 |
| 136.6 | ± | 5.5 | 24.9 |
| 164.3 | ± | 0.4 | 51.5 |
| 184.5 | ± | 13.7 | 95.6 |
|  |  |  |  |
| R2-011 | | | |
| Conc. (mg/ml) | | | Viscosity (cP) |
| 14.8 | ± | 0.3 | 1.5 |
| 34.8 | ± | 3.2 | 2.2 |
| 59.0 | ± | 0.5 | 4.4 |
| 68.8 | ± | 2.2 | 5.7 |
| 84.1 | ± | 0.2 | 10.6 |
| 124.3 | ± | 2.6 | 20.9 |
| 168.5 | ± | 4.9 | 30.9 |
|  |  |  |  |
| R2-012 | | | |
| Conc. (mg/ml) | | | Viscosity (cP) |
| 27.0* |  |  | 2.5 |
| 52.9* |  |  | 2.9 |
| 69.7* |  |  | 3.8 |
| 89.1* |  |  | 5.0 |
| 98.4* |  |  | 7.2 |
| 117.6* |  |  | 12.7 |
| 132.0* |  |  | 20.9 |
| 139.1* |  |  | 24.3 |
| 152.4* |  |  | 42.7 |
| 160.3* |  |  | 52.9 |
| 168.1* |  |  | 74.9 |
| 186.2* |  |  | 85.3 |
| 186.2* |  |  | 81.1 |
|  |  |  |  |
| R2-013 | | | |
| Conc. (mg/ml) | | | Viscosity (cP) |
| 17.6 | ± | 6.6 | 2.6 |
| 38.1 | ± | 0.5 | 4.5 |
| 77.8 | ± | 5.6 | 4.9 |
| 117.2 | ± | 11.7 | 8.1 |
| 139.2 | ± | 0.8 | 16.5 |
| 147.1 | ± | 1.4 | 27.7 |
| 187.3 | ± | 9.1 | 91.4 |
|  |  |  |  |
| R2-014 | | | |
| Conc. (mg/ml) | | | Viscosity (cP) |
| 31.7* |  |  | 2.9 |
| 58.0* |  |  | 3.2 |
| 77.0* |  |  | 5.2 |
| 93.9* |  |  | 7.9 |
| 103.1* |  |  | 11.4 |
| 124.7* |  |  | 23.5 |
| 132.1* |  |  | 31.9 |
| 146.9* |  |  | 48.5 |
| 153.5* |  |  | 58.7 |
| 165.9* |  |  | 78.8 |
| 187.0* |  |  | 158.4 |
|  |  |  |  |
| R2-015 | | | |
| Conc. (mg/ml) | | | Viscosity (cP) |
| 28.0 | ± | 0.1 | 3.2 |
| 55.7 | ± | 0.3 | 4.3 |
| 77.1 | ± | 0.1 | 6.8 |
| 102.3 | ± | 1.2 | 14.5 |
| 120.4 | ± | 0.5 | 30.9 |
| 129.8 | ± | 2.3 | 46.4 |
| 142.7 | ± | 0.5 | 62.0 |
| 154.5 | ± | 0.6 | 94.5 |
| 159.7 | ± | 0.2 | 120.9 |
|  |  |  |  |
| R2-016 | | | |
| Conc. (mg/ml) | | | Viscosity (cP) |
| 25.7 | ± | 0.1 | 2.9 |
| 51.1 | ± | 0.9 | 4.0 |
| 72.3 | ± | 1.1 | 6.0 |
| 89.4 | ± | 1.3 | 9.6 |
| 103.0 | ± | 0.8 | 13.9 |
| 119.9 | ± | 1.9 | 28.0 |
| 132.7 | ± | 2.1 | 37.3 |
| 141.6 | ± | 1.9 | 50.3 |
| 146.7 | ± | 2.1 | 59.8 |
|  |  |  |  |
| R2-017 | | | |
| Conc. (mg/ml) | | | Viscosity (cP) |
| 27.9 | ± | 0.3 | 2.6 |
| 52.4 | ± | 0.3 | 3.7 |
| 72.6 | ± | 0.6 | 5.6 |
| 88.5 | ± | 0.1 | 8.4 |
| 108.3 | ± | 0.7 | 12.9 |
| 130.0 | ± | 1.3 | 27.3 |
| 137.6 | ± | 0.7 | 38.1 |
| 142.8 | ± | 1.7 | 57.2 |
| 154.2 | ± | 0.1 | 109.6 |
|  |  |  |  |
| R2-018 | | | |
| Conc. (mg/ml) | | | Viscosity (cP) |
| 27.5 | ± | 1.9 | 2.8 |
| 33.3 | ± | 1.0 | 2.9 |
| 54.3 | ± | 1.6 | 3.6 |
| 118.4 | ± | 1.6 | 10.8 |
| 150.1 | ± | 2.1 | 18.5 |
| 171.7 | ± | 4.6 | 140.5 |
|  |  |  |  |
| R2-019 | | | |
| Conc. (mg/ml) | | | Viscosity (cP) |
| 15.2* |  |  | 1.8 |
| 40.3 | ± | 0.5 | 3.1 |
| 67.7 | ± | 0.5 | 11.4 |
| 73.2* |  |  | 18.8 |
| 118.2* |  |  | 29.9 |
| 163.2 | ± | 0.6 | 74.1 |
|  |  |  |  |
| R2-020 | | | |
| Conc. (mg/ml) | | | Viscosity (cP) |
| 24.9 | ± | 0.0 | 1.5 |
| 50.8 | ± | 0.3 | 2.0 |
| 70.8 | ± | 0.7 | 2.6 |
| 85.3 | ± | 0.1 | 3.2 |
| 101.5 | ± | 0.3 | 4.2 |
| 121.7 | ± | 0.1 | 6.2 |
| 132.3 | ± | 1.4 | 7.3 |
| 141.4 | ± | 1.4 | 9.0 |
| 155.8 | ± | 1.2 | 10.9 |
| 167.1 | ± | 1.6 | 13.9 |
| 200.5 | ± | 2.5 | 30.7 |
|  |  |  |  |
| R2-021 | | | |
| Conc. (mg/ml) | | | Viscosity (cP) |
| 24.8 | ± | 0.2 | 2.5 |
| 52.5 | ± | 0.1 | 4.3 |
| 71.3 | ± | 0.4 | 7.4 |
| 89.0 | ± | 0.6 | 12.7 |
| 102.4 | ± | 0.9 | 21.5 |
| 117.4 | ± | 0.3 | 39.0 |
| 133.7 | ± | 0.7 | 61.4 |
| 141.9 | ± | 1.4 | 83.8 |
| 144.1 | ± | 0.8 | 125.4 |
| 161.2 | ± | 0.2 | 163.9 |
| 174.0 | ± | 3.2 | 259.0 |
|  |  |  |  |
| R2-022 | | | |
| Conc. (mg/ml) | | | Viscosity (cP) |
| 24.2 | ± | 0.2 | 2.3 |
| 48.2 | ± | 0.2 | 3.8 |
| 69.4 | ± | 0.5 | 7.1 |
| 88.3 | ± | 1.0 | 14.0 |
| 98.8 | ± | 0.2 | 20.8 |
| 129.6 | ± | 0.1 | 69.5 |
| 140.4 | ± | 0.2 | 97.3 |
| 149.2 | ± | 0.9 | 132.2 |
| 160.8 | ± | 0.8 | 194.6 |
